# Pubertal Timing Associates With Cardiometabolic Markers During Puberty and in Young Adulthood

**DOI:** 10.1111/apa.70624

**Published:** 2026-05-29

**Authors:** Saga Nummela, Katja Pahkala, Sinikka Karppinen, Jorma Toppari, Olli Raitakari, Jorma Viikari, Tapani Rönnemaa, Harri Niinikoski

**Affiliations:** ^1^ University of Turku Turku Finland; ^2^ The Wellbeing Service County of Southwest Finland Turku Finland; ^3^ Centre for Population Health Research, University of Turku and Turku University Hospital Turku Finland; ^4^ Research Centre of Applied and Preventive Cardiovascular Medicine, University of Turku Turku Finland; ^5^ Unit of Health and Physical Activity Paavo Nurmi Centre, University of Turku Turku Finland; ^6^ Department of Pediatrics and Adolescent Medicine Turku University Hospital and University of Turku Turku Finland; ^7^ InFLAMES Research Flagship, University of Turku Turku Finland; ^8^ Institute of Biomedicine, Research Centre for Integrative Physiology and Pharmacology, University of Turku Turku Finland; ^9^ Department of Pediatrics and Adolescent Medicine Turku University Hospital Turku Finland; ^10^ Department of Clinical Physiology and Nuclear Medicine Turku University Hospital Turku Finland; ^11^ Division of Medicine Turku University Hospital Turku Finland; ^12^ Department of Medicine University of Turku Turku Finland; ^13^ Division of Medicine, Department of Medicine Turku University Hospital, University of Turku Turku Finland

**Keywords:** blood pressure, body mass index, cardiometabolic health, puberty, serum lipids

## Abstract

**Aim:**

To study how pubertal timing associates with cardiometabolic measures in puberty and young adulthood.

**Methods:**

Cardiovascular risk factors, anthropometry and pubertal development were regularly studied in STRIP study subjects until age 26 years. The study participants were divided into tertiles (early, average, and late) according to the age at the beginning of breast/testicular growth, menarche in females, and end of testicular growth in males. Differences in cardiometabolic variables between tertiles were analysed using one‐way ANOVA.

**Results:**

Earlier breast development was associated with higher body mass index (BMI), waist circumference (WC), and blood pressure (BP) in adulthood compared to the late group. Earlier age at menarche was associated with higher BMI and WC in adulthood. Earlier beginning of testicular enlargement was associated with higher insulin and insulin resistance at 26 years when compared to the late group. Earlier end of testicular growth tended to have higher insulin values and insulin resistance at 26 years than the average group.

**Conclusions:**

Early pubertal development in females is associated with higher BMI and WC. In males, early pubertal development is associated with increased insulin levels and insulin resistance. More research is needed to study whether these metabolic differences build up a cumulative burden.

## Summary

- Early puberty has been associated with increased cardiometabolic risk later in life, but it remains unclear when these cardiometabolic changes can be seen.
- Earlier puberty in females is associated with higher body mass index and waist circumference before, during, and after puberty, while in males, earlier puberty is associated with higher insulin resistance in adulthood.
- These findings improve our understanding of how pubertal timing may reflect later disease burden.

**Abbreviations**
B2: beginning of breast development
BMI: body mass index
BP: blood pressure
CRP: C‐reactive protein
G2: beginning of testicular development
G5: end of testicular development
GlycA: glycoprotein acetyls
HDL‐C: high‐density lipoprotein cholesterol
HOMA‐IR: homeostatic model assessment of Insulin Resistance
IQR: interquartile range
LDL‐C: low‐density lipoprotein cholesterol
M: menarche
SD: standard deviations
STRIP: the Special Turku Coronary Risk Factor Intervention Project
TC: total cholesterol
TG: triglycerides
WC: waist circumference

## 1 Introduction

During the last decades pubertal timing has shifted to an earlier age in both males and females [1]. Concurrently, childhood obesity rates have also been increasing [1, 2], and increased adiposity in children has indeed been associated with earlier pubertal development in both males and females [3].

Puberty begins with the start of breast growth in females and testicular growth in males [4, 5, 6]. For females an apparent culmination of puberty is menarche [4]. There is no corresponding milestone for males, which is why the end of testicular enlargement according to Tanner stages can be considered as the end of pubertal development [5].

The effects of sex hormones in puberty do not only develop secondary sex characteristics but they also cause changes in cardiometabolic measures [7]. Insulin resistance increases at the onset of puberty but returns to baseline by the end of puberty [8]. Blood pressure increases and reaches adult levels by the end of puberty [9]. There are diverse changes in lipid metabolism, depending on both age and pubertal stage [10].

Early puberty has been associated with increased cardiometabolic risk and other non‐communicable diseases later in life [11]. Most convincingly it has been associated with increased adult Body Mass Index (BMI) [11, 12], especially in females with early menarche [12]. Earlier pubertal development has also been associated with a disadvantageous glucose metabolism later in life [11, 13]. In some studies, earlier pubertal timing has been associated with higher blood pressure (BP) [14]. Although some findings indicate an association between pubertal timing and high‐density lipoprotein cholesterol (HDL‐C) and triglycerides (TG) [15], the association between pubertal timing and adult lipid values remains uncertain, with no convincing evidence for an association with total cholesterol (TC) or low‐density lipoprotein cholesterol (LDL‐C) [12]. Inflammatory biomarkers, such as C‐reactive protein (CRP), have been associated with later cardiometabolic diseases [16]. These inflammatory biomarkers seem to be affected by sex hormones and thus by pubertal stage [17]. A novel inflammation marker is Glycoprotein acetyls (GlycA), which has been reported to predict cardiovascular morbidity and mortality [18].

Although we have plenty of evidence of how early puberty is associated with cardiometabolic markers in puberty and in adulthood, there is a lack of knowledge whether these metabolic differences begin before puberty, if they persist to adulthood, or whether they possibly build up a cumulative burden by adulthood. The aim of this study was to investigate how pubertal timing is associated with cardiometabolic markers prepubertally at 7 years of age, at pubertal milestones, at 20 and 26 years of age by leveraging the longitudinal data from the Special Turku Coronary Risk Factor Intervention Project (STRIP).

## 2 Materials and Methods

### 2.1 STRIP Study Design

The STRIP study is an infancy onset prospective intervention study with the aim to prevent atherosclerosis risk‐factors. Recruitment of 5‐month‐old infants and their families began at well‐baby clinics in Turku, Finland, at the beginning of March 1989 and continued to the end of June 1990. Initially, 1105 families were interested to participate in the study and finally 1062 infants and their families were randomly allocated to the intervention group (*n* = 540) or the control group (*n* = 522) [19].

The intervention group received dietary counselling twice a year from 7 months to 20 years of age. The control group met study personnel from 7 months to 7 years of age twice a year, and thereafter once a year to the age of 20 years. The intervention was based on the current Nordic Dietary Recommendations. The main aim of the intervention was to replace saturated fat with unsaturated fat. Decreasing total fat consumption was not a target. A secondary aim was to increase the consumption of fruit, vegetable, and whole‐grain products. The control group received basic health education given at Finnish well‐baby clinics and in school healthcare. Both groups came for follow‐up visits at 26 years of age. Throughout the study both groups met the same study personnel and similar measurements were obtained.

The present study combined the data from both the intervention and control group, as we have previously shown that pubertal development in the intervention and control groups was similar [20]. In the current study we included participants, of whom cardiometabolic measures, growth, and pubertal information were available, with the following exclusions. (1) Individuals with known conditions that affect growth and/or pubertal development or cardiometabolic measures were excluded. These were individuals with familial hypercholesterolemia, type 1 diabetes, Down Syndrome, cerebral palsy and other developmental disorders. (2) Inaccuracies in observation of pubertal development and reported menarche led to exclusions. These were 26 females, where the pubertal duration came out to be < 0 year, and 3 males, whose pubertal duration came out to be < 1 year. After 34 exclusions and 515 lost to follow up, data of 278 females and 235 males were used in the final analyses.

The study protocol was approved by the local ethics committee on 7 November 1989, and the guidelines of the Declaration of Helsinki were followed accordingly. Informed consent was obtained from the parents at the beginning of the study and from the participants at ages 15 years and 18 years [21].

### 2.2 Measurements of Growth, Blood Pressure and Pubertal Stage

Height, weight, waist circumference (WC) and BP were measured at each study visit. Height was measured with a Harpenden stadiometer (Holtain, Crymych, U.K) to the nearest 0.1 cm. Measurements of weight, with participants wearing light clothing, were made with an electronic scale (S10, Soehnle, Murrhardt, Germany) to the nearest 0.1 kg. BMI was calculated as weight in kilograms divided by height in meters squared (kg/m2). WC was measured to the nearest 0.5 cm midway between the iliac crest and the lowest rib at the midaxillary line using a flexible measuring tape. BP was measured after > 15 min rest with an oscillometric noninvasive blood pressure monitor (Criticon Dinamap Compact T). An accurate cuff size was chosen according to the subject's right arm. The average of two measurements was used in statistical analyses [21].

Pubertal development was evaluated by study personnel from 9 years of age onward at each study visit. Breast development (B) was assessed by palpation and breast diameter was measured by a ruler [4]. Testicular development (G) was evaluated visually and by measuring the length of the testis by a ruler [5, 22]. Age at menarche was self‐reported with monthly accuracy. The beginning of puberty was estimated to be the first assessment of Tanner stage ≥ B2 in girls and ≥ G2 (= testis length > 25 mm) in boys. The end of puberty was set as the age of menarche (M) for girls and the end of testicular growth (G5) for boys [5].

### 2.3 Laboratory Methods

Lipid measures were obtained at annual study visits, excluding ages 6 and 8 years, and at the 20‐year and 26‐year study visits. For lipid analyses the blood samples were low speed centrifuged after clotting at room temperature and the separated serum was frozen at −25°C until analysed. TC and TG were measured using a fully enzymatic cholesterol oxidase‐p‐aminophenazone method by an automatic Olympus AU400 analyser (Merck, Darmstadt, Germany) [21]. HDL‐C concentration was analysed after precipitation of non‐HDL lipoproteins with dextran sulfate 500 000. LDL‐C concentration was calculated using the Friedwald formula [21]. If levels of TG were ≥ 4.25 mmol/L (> 400 mg/dL) LDL‐C was set as missing.

In a time‐restricted subsample, consisting of 100 females and 100 males equally from the intervention and control groups, serum glucose and insulin measures were obtained at 7, 9, 11 and 13 years of age. After 15 years of age serum glucose and insulin measures were obtained from all participants annually. Measurements of serum glucose were made using a hexokinase method (Glucose Olympus System Reagent, Olympus, Ireland, Interassay CV 1.8%). For insulin analyses the blood samples were centrifuged immediately and 30 μL Trasylol was added to the 0.5‐mL serum sample prior to freezing until analysis. Serum insulin was measured by a radioimmunoassay (Pharmacia Diagnostics, Uppsala, Sweden) [21]. Homeostatic Model assessment of Insulin Resistance (HOMA‐IR) was calculated (fasting insulin mU/L’×’fasting glucose mmol/L/22.5) to estimate insulin sensitivity [23].

CRP was measured at ages 11, 13, 15, 17, 18, 19, 20, and 26 years. Serum CRP was analysed using a turbidimetric immunoassay kit (‘CRP‐UL’‐assay, Wako Chemicals, Neuss, Germany) with an automated analyser (Olympus AU400) [24]. GlycA was measured at ages 9, 11, 13, 15, 17, 19, and 26 years. GlycA was analysed as part of a nuclear magnetic resonance metabolomics platform (Nightingale Health, Helsinki, Finland) [25]. As CRP and GlycA were only measured at selected ages during the study, we chose to only include CRP in analyses at 20‐ and 26‐years, and GlycA at only 26‐years. At pubertal milestones the number of subjects with measurements of CRP and GlycA were missing, small, and uneven between the groups.

Blood samples were drawn after an overnight fast after 5 years of age. All analyses were performed at the laboratory of the National Public Health Institute in Turku, Finland [21].

### 2.4 Statistical Analyses

Distribution of the continuous variables was evaluated visually and using the Kolmogorov–Smirnov test. Normally distributed variables were presented using mean values, standard deviations (SD), and minimum–maximum range. Variables with skewed distributions were logarithm converted for analyses and were presented using median, Interquartile range (IQR), and minimum–maximum range.

To study how pubertal timing is associated with cardiometabolic measures, we divided each pubertal milestone into three groups as equal in size as possible. The groups were referred to as early, average, and late. Age at B2 in the early group was 8–10 years (37.2%), in the average group 11 years (26.8%), and in the late group 12–16 years (36.0%). Age at menarche was divided exactly into tertiles; age at menarche in the M_early_ group 10–12.3 years, M_average_ 12.4–13.4 years, and M_late_ 13.5–17 years. Age at G2 in the early group was 10–11 years (19.4%), in the average group 12 years (44.9%), and in the late group 13–17 years (35.7%). Age at G5 in G5_early_ was 13–15 years (24.9%), in G5_average_ 16 years (47.4%), and in G5_late_ 17–20 years (27.6%). Distribution of subjects belonging to intervention versus control group were quite equal in all pubertal groups.

Differences between groups were analysed using one‐way analysis of variance (ANOVA) comparing the means of the cardiometabolic variable between the subgroups prepubertally at 7 years of age, at each pubertal milestone, and at 20 and 26 years of age. Tukey's HSD Test for multiple comparisons was used for subgroup analyses.

All statistical analyses were conducted using SAS statistical software, release 9.4 (SAS institute inc., North Carolina, USA). For results, *p*‐values < 0.05 were considered statistically significant.

## 3 Results

For both males and females, cardiometabolic measures were normally distributed at all measuring points, except for TG, insulin, HOMA‐IR, and CRP (Tables [Link], [Link]).

### 3.1 Females

#### 3.1.1 Beginning of Breast Development

Before puberty, at 7 years of age, BMI and WC were higher in females with early (B2_early_) and average (B2_average_) onset of puberty than in those with late onset of puberty (B2_late_). Females with earlier (early < average < late) onset of puberty were taller at 7 years of age. B2_early_ had higher systolic BP than B2_average_.

At the onset of puberty B2_early_ and B2_average_had higher BMI B2_late_. The subjects in the B2_early_ group were also shorter and had higher levels of TC, LDL‐C, TG, and glucose than B2_late_. Additionally, B2_early_ had higher concentrations of LDL‐C and glucose than B2_average_. The subjects in the B2_average_ group were also shorter and had higher BMI and TG concentrations than B2_late_.

At 20 years of age, both B2_early_ and B2_average_ had significantly higher BMI and larger WC than B2_late_. The B2_early_ group had significantly higher diastolic BP than B2_late_, and B2_average_ had significantly higher CRP than B2_late_.

**FIGURE 1:**
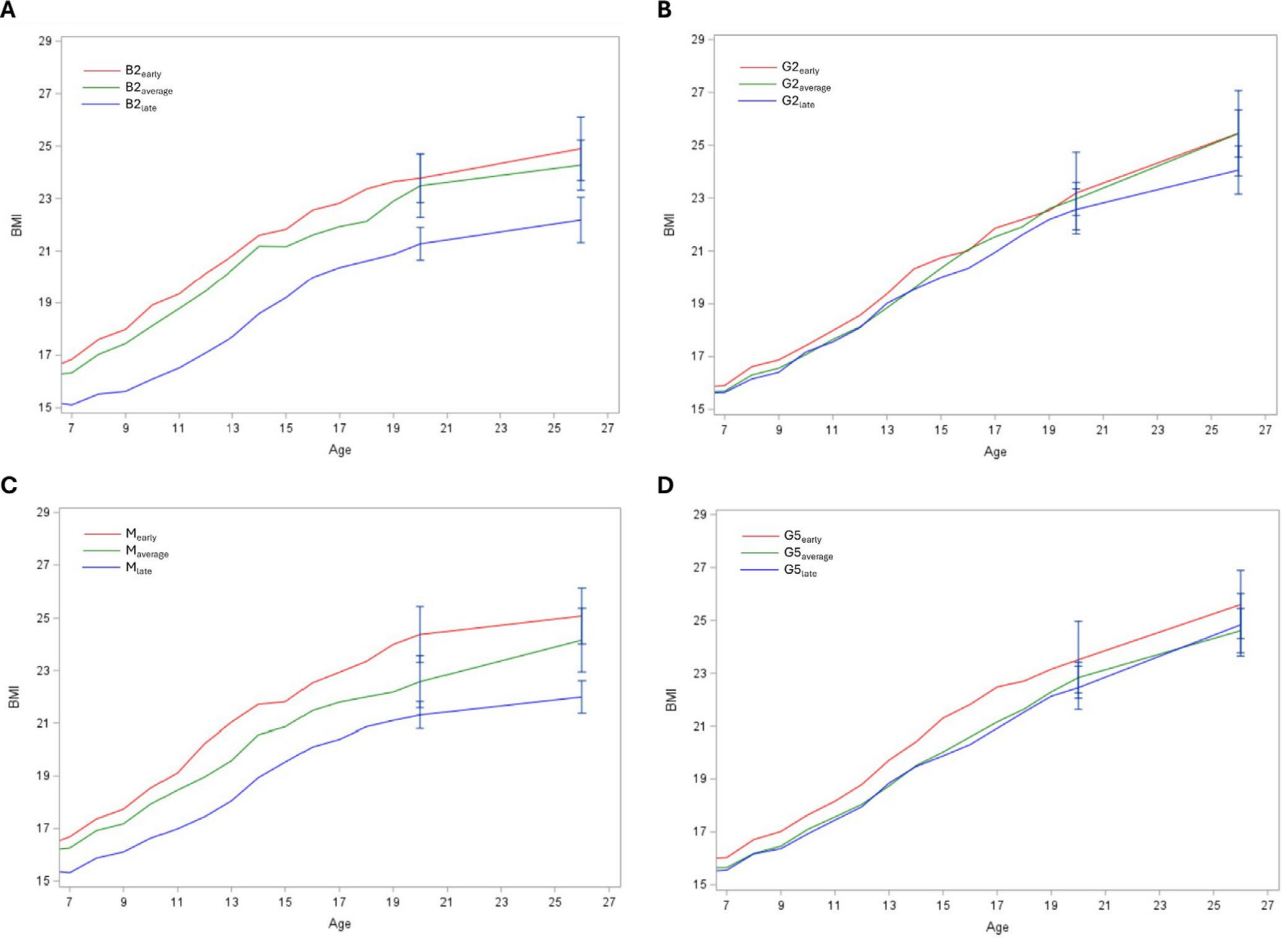
Visual presentation of mean BMI by group. (A) Beginning of breast development (B2) early, average, late (B) Beginning of testicular development (G2) early, average, late (C) Menarche (M) early, average, late (D) End of testicular development (G5) early, average, late. Differences between groups at the pubertal age seen in tables may not be easily interpreted in graphs as ages differ.

**FIGURE 2:**
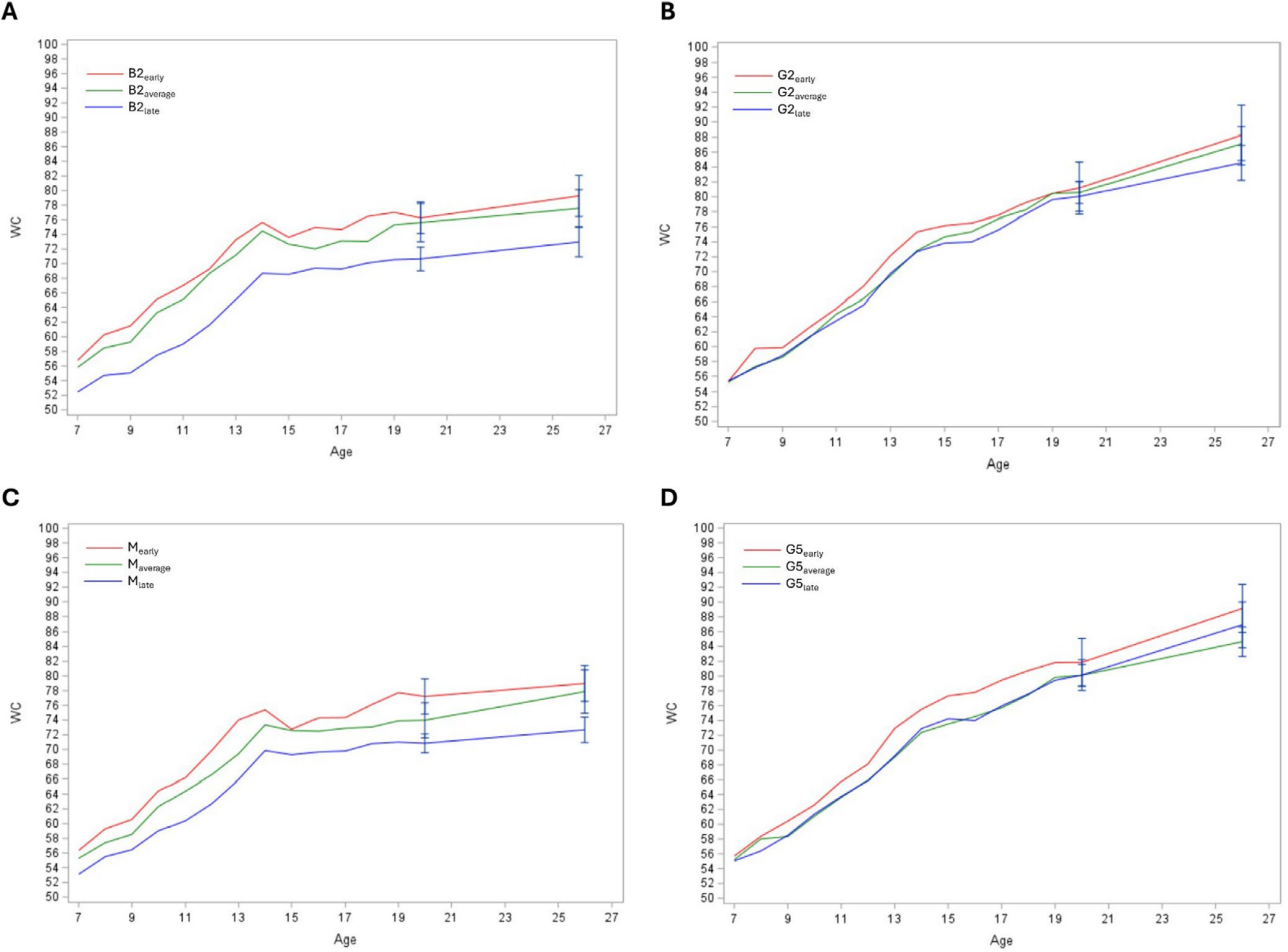
Visual presentation of mean waist circumference (WC) by group. (A) Beginning of breast development (B2) early, average, late (B) Beginning of testicular development (G2) early, average, late (C) Menarche (M) early, average, late (D) End of testicular development (G5) early, average, late. Differences between groups at the pubertal age seen in tables may not be easily interpreted in graphs as ages differ.

**FIGURE 3:**
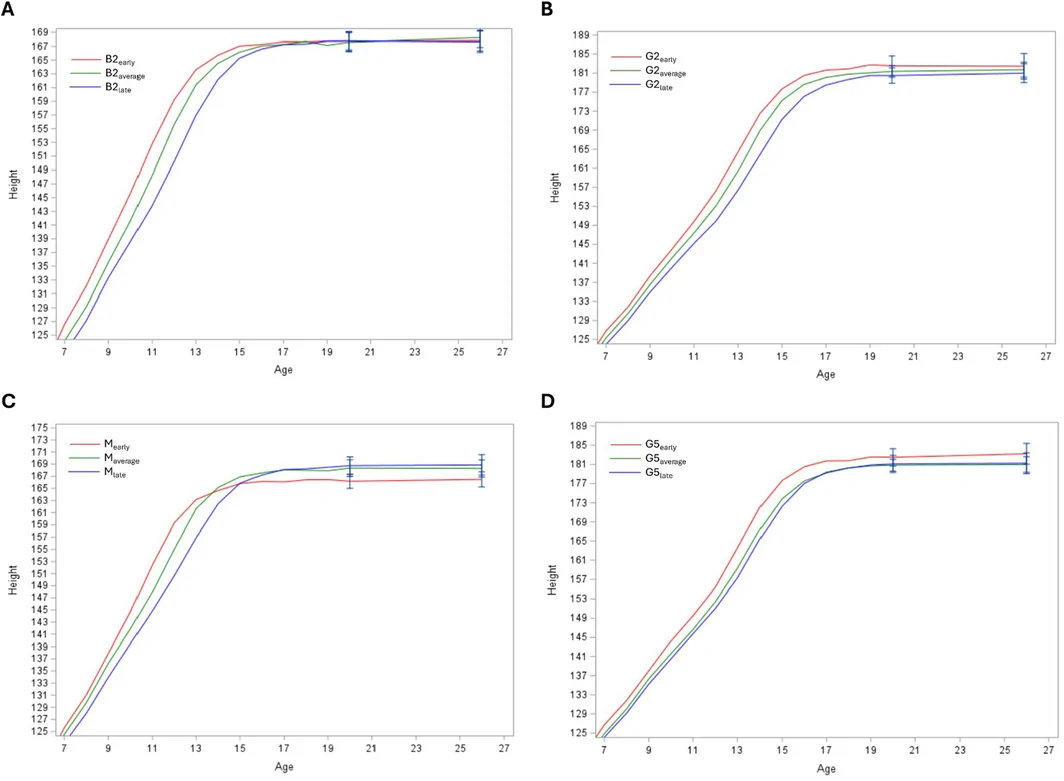
Visual presentation of mean height by group. (A) Beginning of breast development (B2) early, average, late (B) Beginning of testicular development (G2) early, average, late (C) Menarche (M) early, average, late (D) End of testicular development (G5) early, average, late. Differences between groups at the pubertal age seen in tables may not be easily interpreted in graphs as ages differ.

At 26 years of age, both B2_early_ and B2_average_ had higher BMI and larger WC compared to B2_late_. The B2_early_ group had significantly higher diastolic and systolic BP than B2_late_ (Table 1, Figures 1, 2, 3, S1, S3, and S4).

**TABLE 1:** Differences between groups of beginning of breast development (B2).

	7 years			B2			20 years			26 years		
	*p*	Difference in means^a^	CI 95%	*p*	Difference in means^a^	CI 95%	*p*	Difference in means^a^	CI 95%	*p*	Difference in means^a^	CI 95%
BMI (kg/m^2^)	**All** < **0.001**			**All** < **0.001**			**All** < **0.001**			**All < 0.001**		
B2_early_ versus B2_average_	0.21	0.51	−0.21 to 1.23	0.94	−0.14	−1.12 to 0.83	0.90	0.29	−1.28 to 1.86	0.67	0.63	−1.12 to 2.37
B2_early_ versus B2_late_	**< 0.001**	**1.73**	**1.06 to 2.41**	**0.002**	**1.35**	**0.44 to 2.26**	**< 0.001**	**2.50**	**1.03 to 3.97**	**< 0.001**	**2.72**	**1.03 to 4.40**
B2_average_ versus B2_late_	**0.003**	**1.22**	**0.49 to 1.95**	**0.001**	**1.49**	**0.51 to 2.47**	**0.003**	**2.21**	**0.62 to 3.80**	**0.02**	**2.09**	**0.33 to 3.85**
Height (cm)	**All < 0.001**			**All < 0.001**			All 0.98			All 0.84		
B2_early_ versus B2_average_	**0.002**	**2.43**	**0.72 to 4.14**	**< 0.001**	**−6.16**	**−6.78 to −5.55**	0.99	0.14	−2.20 to 2.48	0.91	−0.44	−2.94 to 2.06
B2_early_ versus B2_late_	**< 0.001**	**4.30**	**2.69 to 5.90**	**< 0.001**	**−10.69**	**−11.25 to −10.13**	0.99	−0.06	−2.25 to 2.12	0.98	0.17	−2.24 to 2.59
B2_average_ versus B2_late_	**0.03**	**1.87**	**0.13 to 3.60**	**< 0.001**	**−4.53**	**−5.14 to −3.91**	0.98	−0.20	−2.57 to 2.16	0.83	0.61	−1.91 to 3.13
WC (cm)	**All < 0.001**			All 0.17			**All < 0.001**			**All 0.001**		
B2_early_ versus B2_average_	0.42	0.97	−0.85 to 2.79	0.41	−1.39	−3.93 to 1.16	0.90	0.67	−2.97 to 4.30	0.61	1.70	−2.52 to 5.90
B2_early_ versus B2_late_	**< 0.001**	**4.33**	**2.68 to 5.98**	0.81	0.62	−1.76 to 3.00	**< 0.001**	**5.63**	**2.22 to 9.04**	**< 0.001**	**6.32**	**2.26 to 10.37**
B2_average_ versus B2_late_	**< 0.001**	**3.37**	**1.53 to 5.20**	0.15	2.00	−0.54 to 4.55	**0.005**	**4.96**	**1.27 to 8.65**	**0.03**	**4.62**	**0.39 to 8.85**
Systolic BP (mmHg)	**All 0.01**			All 0.98			All 0.22			**All 0.02**		
B2_early_ versus B2_average_	**0.01**	**4.32**	**0.93 to 7.70**	0.98	0.30	−3.05 to 3.65	0.47	2.37	−2.42 to 7.16	0.13	3.00	−0.64 to 6.63
B2_early_ versus B2_late_	0.14	2.59	−0.60 to 5.78	0.99	0.22	−2.91 to 3.34	0.22	3.18	−1.30 to 7.65	**0.02**	**4.10**	**0.59 to 7.61**
B2_average_ versus B2_late_	0.46	−1.73	−5.16 to 1.71	0.99	−0.09	−3.44 to 3.27	0.92	0.81	−4.03 to 5.65	0.76	1.10	−2.56 to 4.76
Diastolic BP (mmHg)	All 0.97			All 0.34			**All 0.04**			**All 0.02**		
B2_early_ versus B2_average_	0.98	0.17	−2.12 to 2.46	0.42	1.20	−1.05 to 3.46	0.67	1.08	−1.92 to 4.08	0.75	0.82	−1.86 to 3.51
B2_early_ versus B2_late_	0.98	0.20	−1.96 to 2.35	0.42	1.12	−0.98 to 3.22	**0.03**	**3.06**	**0.25 to 5.86**	**0.02**	**3.05**	**0.46 to 5.46**
B2_average_ versus B2_late_	0.99	0.03	−2.30 to 3.35	0.99	−0.08	−2.34 to 2.18	0.28	1.98	−1.06 to 5.00	0.13	2.23	−0.47 to 4.93
TC (mmol/L)	All 0.39			**All 0.02**			All 0.92			All 0.59		
B2_early_ versus B2_average_	0.37	0.15	−0.11 to 0.42	0.11	0.23	−0.04 to 0.51	0.94	−0.04	−0.37 to 0.28	0.58	−0.15	−0.51 to 0.21
B2_early_ versus B2_late_	0.91	0.04	−0.21 to 0.30	**0.01**	**0.30**	**0.05 to 0.56**	0.99	0.003	−0.30 to 0.31	0.78	−0.10	−0.44 to 0.25
B2_average_ versus B2_late_	0.61	−0.11	−0.38 to 0.16	0.83	0.07	−0.20 to 0.34	0.94	0.05	−0.28 to 0.37	0.94	0.05	−0.31 to 0.41
HDL‐C (mmol/L)	All 0.72			All 0.88			All 0.62			All 0.76		
B2_early_ versus B2_average_	0.94	0.01	−0.07 to 0.09	0.99	−0.002	−0.09 to 0.09	0.91	−0.02	−0.15 to 0.10	0.74	−0.04	−0.17 to 0.09
B2_early_ versus B2_late_	0.87	−0.02	−0.09 to 0.06	0.89	−0.02	−0.10 to 0.06	0.83	0.03	−0.09 to 0.15	0.89	−0.02	−0.15 to 0.10
B2_average_ versus B2_late_	0.71	−0.03	−0.11 to 0.05	0.92	−0.01	−0.10 to 0.07	0.60	0.05	−0.08 to 0.18	0.96	0.02	−0.11 to 0.15
LDL‐C (mmol/L)	All 0.40			**All 0.01**			All 0.95			All 0.41		
B2_early_ versus B2_average_	0.37	0.14	−0.10 to 0.37	**0.04**	**0.25**	**0.01 to 0.50**	0.99	−0.01	−0.29 to 0.26	0.44	−0.15	−0.45 to 0.14
B2_early_ versus B2_late_	0.85	0.05	−0.17 to 0.28	**0.02**	**0.26**	**0.03 to 0.49**	0.94	−0.04	−0.29 to 0.22	0.55	−0.13	−0.41 to 0.16
B2_average_ versus B2_late_	0.68	−0.09	−0.33 to 0.16	0.99	0.01	−0.23 to 0.25	0.98	−0.02	−0.30 to 0.26	0.97	−0.03	−0.27 to 0.32
TG^b^ (mmol/L)	All 0.89			**All 0.01**			All 0.80			All 0.55		
B2_early_ versus B2_average_	0.96	0.02	−0.12 to 0.15	0.80	−0.04	−0.19 to 0.11	0.98	−0.01	−0.19 to 0.16	0.67	0.06	−0.11 to 0.24
B2_early_ versus B2_late_	0.88	0.03	−0.10 to 0.15	**< 0.05**	**0.14**	**0.0001 to 0.27**	0.87	0.03	−0.13 to 0.20	0.57	0.07	−0.10 to 0.24
B2_average_ versus B2_late_	0.98	0.01	−0.12 to 0.14	**0.01**	**0.18**	**0.03 to 0.32**	0.80	0.05	−0.13 to 0.22	0.99	0.01	−0.17 to 0.18
Glucose (mmol/L)	—			**All < 0.001**			All 0.09			All 0.82		
B2_early_ versus B2_average_	—	—	—	**< 0.001**	**−0.31**	**−0.49 to −0.14**	0.11	−0.14	−0.30 to 0.02	0.97	−0.02	−0.21 to 0.17
B2_early_ versus B2_late_	—	—	—	**0.004**	**−0.31**	**−0.53 to −0.09**	0.20	−0.11	−0.26 to 0.04	0.92	0.03	−0.15 to 0.21
B2_average_ versus B2_late_	—	—	—	0.99	0.002	−0.20 to 0.20	0.93	0.03	−0.14 to 0.19	0.81	0.05	−0.14 to 0.24
Insulin^b^ (mU/L)	—			All 0.10			All 0.59			All 0.69		
B2_early_ versus B2_average_	—	—	—	0.10	−0.26	−0.56 to 0.04	0.60	−0.07	−0.25 to 0.10	0.68	0.07	−0.12 to 0.25
B2_early_ versus B2_late_	—	—	—	0.45	−0.21	−0.62 to 0.20	0.99	−0.01	−0.17 to 0.16	0.82	0.05	−0.13 to 0.23
B2_average_ versus B2_late_	—	—	—	0.95	0.05	−0.37 to 0.48	0.69	0.06	−0.12 to 0.24	0.97	−0.02	−0.21 to 0.17
HOMA‐IR^b^	—			All 0.06			All 0.46			All 0.75		
B2_early_ versus B2_average_	—	—	—	0.05	−0.37	−0.75 to 0.001	0.44	−0.10	−0.29 to 0.09	0.77	0.06	−0.14 to 0.26
B2_early_ versus B2_late_	—	—	—	0.23	−0.32	−0.79 to 0.15	0.91	−0.03	−0.21 to 0.15	0.81	0.05	−0.14 to 0.25
B2_average_ versus B2_late_	—	—	—	0.95	0.05	−0.37 to 0.48	0.69	0.07	−0.13 to 0.26	0.99	−0.01	−0.21 to 0.20
CRP^b^ (mg/L)	—			—			**All 0.05**			All 0.23		
B2_early_ versus B2_average_	—	—	—	—	—	—	0.74	−0.15	−0.64 to 0.33	0.65	0.19	−0.31 to 0.68
B2_early_ versus B2_late_	—	—	—	—	—	—	0.18	0.34	−0.12 to 0.80	0.20	0.35	−0.13 to 0.84
B2_average_ versus B2_late_	—	—	—	—	—	—	**0.05**	**0.49**	**0.002 to 0.99**	0.72	0.16	−0.34 to 0.67
GlycA (μmol/L)	—			—			—			All 0.35		
B2_early_ versus B2_average_	—	—	—	—	—	—	—	—	—	0.86	0.02	−0.06 to 0.09
B2_early_ versus B2_late_	—	—	—	—	—	—	—	—	—	0.32	0.04	−0.03 to 0.11
B2_average_ versus B2_late_	—	—	—	—	—	—	—	—	—	0.67	0.03	−0.05 to 0.10

*Note:* **Bolded** = Statistically significant.

^a^ Difference in means between X and Y, comparing to X.

^b^ Logarithm converted.

#### 3.1.2 Menarche M

At 7 years of age, both those with early (M_early_) and average (M_average_) menarche had higher BMI and WC than those with late menarche (M_late_). The subjects in the M_early_ group were taller than those in the M_late_ group.

At menarche, both M_early_ and M_average_ were shorter than M_late_. The subjects in the M_average_ group were also shorter than those in M_late_.

At 20 years of age, M_early_ had higher BMI than M_average_ and M_late_. Additionally, the subjects in the M_early_ group were shorter and had bigger WC compared to M_late_. Group M_average_ had higher insulin and HOMA‐IR than M_late_.

At 26 years of age, both M_early_ and M_average_ had higher BMI and bigger WC compared to M_late_ (Table 2, Figures 1, 2, 3, and S5).

**TABLE 2:** Differences between groups of menarche (M).

	7 years			Menarche			20 years			26 years		
	*p*	Difference in means^a^	CI 95%	*p*	Difference in means^a^	CI 95%	*p*	Difference in means^a^	CI 95%	*p*	Difference in means^a^	CI 95%
BMI (kg/m^2^)	**All < 0.001**			All 0.06			**All < 0.001**			**All < 0.001**		
M_early_ versus M_average_	0.34	0.43	−0.29 to 1.15	0.73	0.32	−0.67 to 1.32	**0.01**	**1.80**	**0.30 to 3.31**	0.41	0.92	−0.78 to 2.62
M_early_ versus M_late_	**< 0.001**	**1.37**	**0.64 to 2.10**	0.049	1.02	0.005 to 2.03	**< 0.001**	**3.07**	**1.57 to 4.57**	**< 0.001**	**3.09**	**1.34 to 4.84**
M_average_ versus M_late_	**0.01**	**0.94**	**0.22 to 1.67**	0.24	0.70	−0.31 to 1.71	0.13	1.27	−0.28 to 2.81	**0.01**	**2.17**	**0.46 to 3.88**
Height (cm)	**All 0.003**			**All < 0.001**			**All 0.01**			All 0.06		
M_early_ versus M_average_	0.30	1.11	−0.64 to 2.85	**< 0.001**	**−3.80**	**−4.46 to −3.14**	0.06	−2.17	−4.39 to 0.04	0.18	−1.80	−4.22 to 0.61
M_early_ versus M_late_	**0.002**	**2.57**	**0.81 to 4.33**	**< 0.001**	**−6.82**	**−7.47 to −6.16**	**0.02**	**−2.58**	**−4.79 to 0.36**	0.06	−2.40	−4.89 to 0.09
M_average_ versus M_late_	0.13	1.46	−0.29 to 3.22	**< 0.001**	**−3.01**	**−3.68 to −2.34**	0.91	−0.40	−2.68 to 1.87	0.83	−0.59	−3.02 to 1.84
WC (cm)	**All 0.001**			All 0.96			**All < 0.001**			**All 0.001**		
M_early_ versus M_average_	0.35	1.07	−0.75 to 2.88	0.96	−0.28	−2.82 to 2.27	0.08	3.24	−0.29 to 6.77	0.80	1.10	−3.01 to 5.20
M_early_ versus M_late_	**< 0.001**	**3.24**	**1.44 to 5.04**	0.98	−0.23	−2.81 to 2.35	**< 0.001**	**6.38**	**2.89 to 9.88**	**0.002**	**6.30**	**2.06 to 10.54**
M_average_ versus M_late_	**0.02**	**2.17**	**0.33 to 4.02**	0.99	0.05	−2.52 to 2.62	0.10	3.14	−0.47 to 6.76	**0.01**	**5.21**	**1.07 to 9.35**
Systolic BP (mmHg)	All 0.61			All 0.16			All 0.30			All 0.65		
M_early_ versus M_average_	0.62	1.32	−2.03 to 4.67	0.33	2.13	−1.40 to 5.66	0.42	2.49	−2.16 to 7.13	0.62	1.42	−2.19 to 5.03
M_early_ versus M_late_	0.99	0.21	−3.16 to 3.59	0.91	−0.64	−4.25 to 2.97	0.35	2.73	−1.89 to 7.36	0.84	0.89	−2.84 to 4.62
M_average_ versus M_late_	0.72	−1.11	−4.48 to 2.26	0.17	−2.77	−6.36 to 0.82	0.99	0.25	−4.51 to 5.00	0.94	−0.53	−4.17 to 3.11
Diastolic BP (mmHg)	All 0.44			All 0.39			All 0.38			All 0.24		
M_early_ versus M_average_	0.74	0.70	−1.53 to 2.93	0.97	−0.22	−2.44 to 2.00	0.48	1.43	−1.50 to 4.37	0.23	1.86	−0.79 to 4.51
M_early_ versus M_late_	0.85	−0.51	−2.75 to 1.73	0.40	−1.25	−3.52 to 1.02	0.43	1.54	−1.38 to 4.47	0.48	1.35	−1.39 to 4.09
M_average_ versus M_late_	0.41	−1.21	−3.45 to 1.03	0.53	−1.03	−3.29 to 1.23	0.99	0.11	−2.90 to 3.12	0.89	−0.51	−3.19 to 2.16
TC (mmol/L)	All 0.23			All 0.14			All 0.99			All 0.57		
M_early_ versus M_average_	0.25	−0.18	−0.44 to 0.08	0.94	−0.03	−0.26 to 0.20	0.99	−0.003	−0.32 to 0.31	0.55	−0.15	−0.50 to 0.19
M_early_ versus M_late_	0.39	−0.15	−0.41 to 0.12	0.15	−0.18	−0.42 to 0.05	0.99	0.01	−0.30 to 0.32	0.92	−0.06	−0.42 to 0.30
M_average_ versus M_late_	0.96	0.03	−0.23 to 0.29	0.27	−0.15	−0.38 to 0.08	0.99	0.01	−0.31 to 0.33	0.80	0.10	−0.26 to 0.45
HDL‐C (mmol/L)	All 0.53			All 0.86			All 0.87			All 0.26		
M_early_ versus M_average_	0.99	0.0003	−0.08 to 0.08	0.84	−0.02	−0.10 to 0.06	0.90	−0.02	−0.15 to 0.10	0.27	−0.08	−0.21 to 0.04
M_early_ versus M_late_	0.59	−0.03	−0.11 to 0.05	0.96	−0.01	−0.09 to 0.07	0.88	−0.03	−0.15 to 0.10	0.40	−0.07	−0.20 to 0.06
M_average_ versus M_late_	0.60	−0.03	−0.11 to 0.05	0.96	0.01	−0.07 to 0.09	0.99	−0.003	−0.13 to 0.12	0.98	0.01	−0.12 to 0.14
LDL‐C (mmol/L)	All 0.20			All 0.06			All 0.95			All 0.76		
M_early_ versus M_average_	0.19	−0.17	−0.40 to 0.06	0.95	−0.03	−0.23 to 0.18	0.95	0.04	−0.23 to 0.30	0.74	−0.09	−0.38 to 0.20
M_early_ versus M_late_	0.45	−0.12	−0.35 to 0.11	0.07	−0.19	−0.40 to 0.01	0.99	0.01	−0.26 to 0.27	0.91	−0.05	−0.35 to 0.25
M_average_ versus M_late_	0.86	0.05	−0.18 to 0.29	0.14	−0.17	−0.37 to 0.04	0.96	−0.03	−0.30 to 0.24	0.95	0.04	−0.25 to 0.33
TG^b^ (mmol/L)	All 0.86			All 0.76			All 0.67			All 0.34		
M_early_ versus M_average_	0.98	−0.01	−0.14 to 0.12	0.74	0.05	−0.10 to 0.19	0.96	−0.02	−0.19 to 0.15	0.90	0.03	−0.14 to 0.20
M_early_ versus M_late_	0.92	0.02	−0.11 to 0.15	0.93	0.02	−0.13 to 0.17	0.81	0.04	−0.13 to 0.21	0.33	0.11	−0.07 to 0.28
M_average_ versus M_late_	0.85	0.03	−0.10 to 0.16	0.92	−0.02	−0.17 to 0.12	0.65	0.06	−0.11 to 0.24	0.56	0.07	−0.10 to 0.25
Glucose (mmol/L)	—			All 0.55			All 0.56			All 0.85		
M_early_ versus M_average_	—	—	—	0.58	0.11	−0.16 to 0.38	0.61	−0.06	−0.22 to 0.09	0.99	0.01	−0.17 to 0.19
M_early_ versus M_late_	—	—	—	0.63	0.08	−0.13 to 0.29	0.99	0.001	−0.16 to 0.16	0.91	−0.03	−0.22 to 0.16
M_average_ versus M_late_	—	—	—	0.95	−0.03	−0.27 to 0.21	0.62	0.06	−0.10 to 0.23	0.84	−0.04	−0.23 to 0.14
Insulin^b^ (mU/L)	—			All 0.07			**All 0.03**			All 0.39		
M_early_ versus M_average_	—	—	—	0.34	−0.25	−0.67 to 0.18	0.66	−0.06	−0.23 to 0.11	0.99	0.004	−0.18 to 0.18
M_early_ versus M_late_	—	—	—	0.66	0.12	−0.21 to 0.45	0.16	0.13	−0.04 to 0.30	0.45	0.10	−0.09 to 0.29
M_average_ versus M_late_	—	—	—	0.06	0.37	−0.01 to 0.74	**0.02**	**0.19**	**0.02 to 0.36**	0.46	0.09	−0.09 to 0.28
HOMA‐IR^b^	—			All 0.10			**All 0.03**			All 0.51		
M_early_ versus M_average_	—	—	—	0.45	−0.22	−0.67 to 0.22	0.61	−0.07	−0.26 to 0.11	0.99	0.01	−0.19 to 0.20
M_early_ versus M_late_	—	—	—	0.62	0.14	−0.21 to 0.49	0.22	0.13	−0.05 to 0.31	0.55	0.09	−0.11 to 0.29
M_average_ versus M_late_	—	—	—	0.09	0.36	−0.04 to 0.76	**0.03**	**0.20**	**0.02 to 0.39**	0.59	0.08	−0.12 to 0.28
CRP^b^ (mg/L)	—			—			All 0.56			All 0.70		
M_early_ versus M_average_	—	—	—	—	—	—	0.66	0.18	−0.30 to 0.65	0.76	0.15	−0.34 to 0.63
M_early_ versus M_late_	—	—	—	—	—	—	0.59	0.20	−0.28 to 0.68	0.73	0.16	−0.34 to 0.67
M_average_ versus M_late_	—	—	—	—	—	—	0.99	0.03	−0.46 to 0.52	0.99	0.02	−0.48 to 0.51
GlycA (μmol/L)	—			—			—			All 0.44		
M_early_ versus M_average_	—	—	—	—	—	—	—	—	—	0.54	0.03	−0.04 to 0.10
M_early_ versus M_late_	—	—	—	—	—	—	—	—	—	0.47	0.04	−0.04 to 0.11
M_average_ versus M_late_	—	—	—	—	—	—	—	—	—	0.99	0.005	−0.07 to 0.08

*Note:* **Bolded** = Statistically significant.

^a^ Difference in means between X and Y, comparing to X.

^b^ Logarithm converted.

### 3.2 Males

#### 3.2.1 Beginning of Testicular Development

At 7 years of age, males with early onset of puberty (G2_early_) were taller than those with late onset of puberty (G2_late_).

Males with average onset of puberty (G2_average_) were shorter and had lower BMI and WC than the G2_late_ group. Males in the G2_early_ group were also shorter and had smaller WC than G2_late_. Group G2_early_ had higher TC and HDL‐C than G2_late_, while G2_average_ only had higher TC than G2_late_.

At 20 years of age, G2_early_ had higher diastolic BP than G2_late_.

At 26 years of age, G2_early_ had higher insulin and HOMA‐IR than G2_late_ (Table 3, Figures 1, 2, 3, S1, S2, and S5).

**TABLE 3:** Differences between groups of beginning of testicular development (G2).

	7 years			G2			20 years			26 years		
	*p*	Difference in means^a^	CI 95%	*p*	Difference in means^a^	CI 95%	*p*	Difference in means^a^	CI 95%	*p*	Difference in means^a^	CI 95%
BMI (kg/m^2^)	All 0.66			**All 0.02**			All 0.63			All 0.08		
G2_early_ versus G2_average_	0.73	0.21	−0.45 to 0.88	0.91	−0.21	−1.42 to 1.01	0.94	0.22	−1.36 to 1.81	0.99	0.01	−1.94 to 1.97
G2_early_ versus G2_late_	0.65	0.26	−0.43 to 0.95	0.05	−1.25	−2.51 to 0.01	0.65	0.62	−1.03 to 2.27	0.22	1.40	−0.58 to 3.38
G2_average_ versus G2_late_	0.98	0.05	−0.51 to 0.60	**0.04**	**−1.04**	**−2.06 to −0.03**	0.75	0.40	−0.89 to 1.69	0.09	1.38	−0.18 to 2.95
Height (cm)	**All 0.004**			**All < 0.001**			All 0.28			All 0.63		
G2_early_ versus G2_average_	0.18	1.47	−0.47 to 3.40	**< 0.001**	**−3.10**	**−3.95 to −2.25**	0.61	1.17	−1.73 to 4.08	0.89	0.72	−2.97 to 4.41
G2_early_ versus G2_late_	**0.003**	**2.82**	**0.80 to 4.83**	**< 0.001**	**−8.36**	**−9.24 to −7.48**	0.25	2.05	−0.98 to 5.08	0.62	1.47	−2.27 to 5.21
G2_average_ versus G2_late_	0.12	1.35	−0.27 to 2.97	**< 0.001**	**−5.26**	**−5.95 to −4.57**	0.65	0.88	−1.48 to 3.24	0.82	0.75	−2.21 to 3.70
WC (cm)	All 0.97			**All < 0.001**			All 0.79			All 0.15		
G2_early_ versus G2_average_	0.99	0.06	−1.69 to 1.80	0.45	−1.81	−5.46 to 1.73	0.92	0.61	−3.20 to 4.43	0.85	1.15	−3.80 to 6.10
G2_early_ versus G2_late_	0.99	−0.09	−1.92 to 1.73	**< 0.001**	**−5.82**	**−9.50 to −2.13**	0.78	1.14	−2.84 to 5.11	0.19	3.71	−1.30 to 8.73
G2_average_ versus G2_late_	0.97	−0.15	−1.61 to 1.32	**0.005**	**−4.00**	**−6.96 to −1.05**	0.92	0.52	−2.57 to 3.62	0.28	2.56	−1.40 to 6.52
Systolic BP (mmHg)	All 0.06			All 0.42			All 0.26			All 0.91		
G2_early_ versus G2_average_	0.57	1.40	−1.84 to 4.63	0.99	0.18	−3.68 to 4.05	0.99	0.08	−5.56 to 5.71	0.98	−0.40	−5.84 to 5.03
G2_early_ versus G2_late_	0.06	3.26	−0.10 to 6.63	0.64	−1.54	−5.55 to 2.47	0.44	3.04	−2.83 to 8.91	0.98	0.41	−5.10 to 5.92
G2_average_ versus G2_late_	0.24	1.87	−0.84 to 4.57	0.42	−1.72	−4.95 to 1.50	0.28	2.96	−1.61 to 7.53	0.90	0.81	−3.54 to 5.16
Diastolic BP (mmHg)	All 0.12			All 0.57			**All 0.02**			All 0.48		
G2_early_ versus G2_average_	0.17	2.23	−0.07 to 5.11	0.54	−0.17	−3.78 to 1.43	0.32	2.25	−1.43 to 5.93	0.46	2.16	−2.11 to 6.42
G2_early_ versus G2_late_	0.14	2.44	−0.56 to 5.45	0.73	−0.87	−3.57 to 1.83	**0.02**	**4.56**	**0.72 to 8.39**	0.60	1.76	−2.56 to 6.09
G2_average_ versus G2_late_	0.98	0.22	−2.20 to 2.63	0.94	0.30	−1.87 to 2.47	0.16	2.31	−0.68 to 5.29	0.96	−0.39	−3.81 to 3.02
TC (mmol/L)	All 0.67			**All 0.01**			All 0.15			All 0.60		
G2_early_ versus G2_average_	0.97	−0.03	−0.31 to 0.25	0.60	0.12	−0.18 to 0.42	0.31	−0.20	−0.53 to 0.12	0.70	−0.16	−0.65 to 0.32
G2_early_ versus G2_late_	0.88	0.06	−0.23 to 0.35	**0.01**	**0.38**	**0.07 to 0.69**	0.99	−0.01	−0.35 to 0.34	0.99	−0.02	−0.51 to 0.47
G2_average_ versus G2_late_	0.64	0.09	−0.15 to 0.33	**0.04**	**0.26**	**0.01 to 0.51**	0.19	0.20	−0.07 to 0.47	0.65	0.14	−0.24 to 0.53
HDL‐C (mmol/L)	All 0.96			**All 0.02**			All 0.63			All 0.72		
G2_early_ versus G2_average_	0.97	0.01	−0.10 to 0.12	0.71	0.04	−0.08 to 0.15	0.99	−0.002	−0.12 to 0.12	0.76	0.05	−0.11 to 0.21
G2_early_ versus G2_late_	0.99	0.003	−0.11 to 0.12	**0.03**	**0.13**	**0.01 to 0.24**	0.78	0.04	−0.09 to 0.16	0.98	0.01	−0.15 to 0.18
G2_average_ versus G2_late_	0.98	−0.01	−0.10 to 0.09	0.08	0.09	−0.01 to 0.18	0.63	0.04	−0.06 to 0.13	0.80	−0.03	−0.16 to 0.09
LDL‐C (mmol/L)	All 0.65			All 0.06			All 0.09			All 0.42		
G2_early_ versus G2_average_	0.84	−0.06	−0.30 to 0.19	0.74	0.08	−1.18 to 0.34	0.17	−0.23	−0.52 to 0.07	0.54	−0.19	−0.62 to 0.24
G2_early_ versus G2_late_	0.98	0.02	−0.23 to 0.27	0.08	0.24	−0.02 to 0.51	0.95	−0.04	−0.35 to 0.27	0.99	−0.03	−0.46 to 0.41
G2_average_ versus G2_late_	0.64	0.08	−0.13 to 0.28	0.17	0.16	−0.05 to 0.38	0.17	0.19	−0.06 to 0.43	0.49	0.16	−0.18 to 0.51
TG^b^ (mmol/L)	All 0.48			All 0.91			All 0.74			All 0.65		
G2_early_ versus G2_average_	0.76	0.05	−0.12 to 0.21	0.98	−0.02	−0.22 to 0.19	0.78	0.06	−0.15 to 0.27	0.64	−0.09	−0.33 to 0.15
G2_early_ versus G2_late_	0.45	0.09	−0.08 to 0.26	0.99	0.01	−0.20 to 0.22	0.98	0.02	−0.20 to 0.24	0.69	−0.08	−0.33 to 0.16
G2_average_ versus G2_late_	0.79	0.04	−0.10 to 0.18	0.91	0.03	−0.14 to 0.20	0.83	−0.04	−0.22 to 0.13	0.99	0.01	−0.18 to 0.20
Glucose (mmol/L)	—			All 0.29			All 0.18			All 0.87		
G2_early_ versus G2_average_	—	—	—	—	—	—	0.70	−0.06	−0.23 to 0.11	0.86	−0.06	−0.33 to 0.21
G2_early_ versus G2_late_	—	—	—	0.29	−0.15	−0.42 to 0.13	0.18	−0.14	−0.32 to 0.04	0.96	−0.03	−0.31 to 0.24
G2_average_ versus G2_late_	—	—	—	—	—	—	0.39	−0.08	−0.22 to 0.06	0.95	0.03	−0.19 to 0.24
Insulin^b^ (mU/L)	—			All 0.56			All 0.80			**All 0.03**		
G2_early_ versus G2_average_	—	—	—	—	—	—	0.89	0.05	−0.19 to 0.28	0.06	0.28	−0.01 to 0.57
G2_early_ versus G2_late_	—	—	—	0.56	−0.08	−0.38 to 0.21	0.78	0.07	−0.17 to 0.31	**0.02**	**0.33**	**0.03 to 0.63**
G2_average_ versus G2_late_	—	—	—	—	—	—	0.96	0.02	−0.17 to 0.21	0.86	0.05	−0.18 to 0.29
HOMA‐IR^b^	—			All 0.48			All 0.83			**All 0.049**		
G2_early_ versus G2_average_	—	—	—	—	—	—	0.83	0.06	−0.18 to 0.29	0.11	0.27	−0.05 to 0.58
G2_early_ versus G2_late_	—	—	—	0.48	−0.11	−0.44 to 0.21	0.84	0.06	−0.19 to 0.30	**0.04**	**0.32**	**0.01 to 0.64**
G2_average_ versus G2_late_	—	—	—	—	—	—	1.00	0.0005	−0.19 to 0.19	0.85	0.06	−0.19 to 0.31
CRP*yy (mg/L)	—			—			All 0.91			All 0.96		
G2_early_ versus G2_average_	—	—	—	—	—	—	0.94	0.08	−0.48 to 0.63	0.96	0.07	−0.53 to 0.67
G2_early_ versus G2_late_	—	—	—	—	—	—	0.99	0.004	−0.58 to 0.59	0.97	0.06	−0.55 to 0.66
G2_average_ versus G2_late_	—	—	—	—	—	—	0.92	−0.07	−0.53 to 0.38	0.99	−0.01	−0.49 to 0.47
GlycA (μmol/L)	—			—			—			All 0.93		
G2_early_ versus G2_average_	—	—	—	—	—	—	—	—	—	0.95	0.01	−0.08 to 0.11
G2_early_ versus G2_late_	—	—	—	—	—	—	—	—	—	0.93	0.02	−0.08 to 0.11
G2_average_ versus G2_late_	—	—	—	—	—	—	—	—	—	0.99	0.003	−0.07 to 0.08

*Note:* **Bolded** = Statistically significant.

^a^ Difference in means between X and Y, comparing to X.

^b^ Logarithm converted.

#### 3.2.2 End of Testicular Growth

At 7 years of age, males with early end of testicular growth (G2_early_) were taller than those with later end of testicular growth (G2_late_).

Both those with early (G5_early_) and average (G5_average_) end of testicular growth were shorter than G5_late_. Group G5_average_ had lower systolic BP, diastolic BP, and TC than G5_late_. The G5_early_ group had lower TG than G5_late_.

At 20 years of age, there were no significant differences between any groups of G5.

At 26 years of age, marginal though insignificant differences in insulin and HOMA‐IR were found between G5_early_ and G5_average_. Both G5_early_ and G5_late_ had higher CRP than G5_average_. Both G5_early_ and G5_late_ had lower GlycA than G5_average_ (Table 4, Figures 3, S1, S4, and S5).

**TABLE 4:** Differences between groups of end of testicular development (G5).

	*p*	Difference in means^a^	CI 95%	G5			20 years			26 years		
				*p*	Difference in means^a^	CI 95%	*p*	Difference in means^a^	CI 95%	*p*	Difference in means^a^	CI 95%
BMI (kg/m^2^)	All 0.22			All 0.33			All 0.30			All 0.43		
G5_early_ versus G5_average_	0.32	0.38	−0.24 to 0.99	0.46	0.63	−0.62 to 1.87	0.53	0.68	−0.81 to 2.16	0.40	0.99	−0.82 to 2.80
G5_early_ versus G5_late_	0.22	0.48	−0.20 to 1.15	0.99	−0.02	−1.40 to 1.37	0.27	1.06	−0.56 to 2.67	0.64	0.76	−1.22 to 2.76
G5_average_ versus G5_late_	0.92	0.10	−0.48 to 0.68	0.41	−0.64	−1.83 to 0.55	0.78	0.38	−0.95 to 1.71	0.95	−0.22	−1.90 to 1.45
Height (cm)	**All 0.01**			**All < 0.001**			All 0.35			All 0.27		
G5_early_ versus G5_average_	0.05	1.83	0.03 to 3.63	0.93	−0.11	−0.83 to 0.61	0.33	1.65	−1.09 to 4.39	0.26	2.24	−1.12 to 5.60
G5_early_ versus G5_late_	**0.01**	**2.59**	**0.60 to 4.58**	**< 0.001**	**−2.06**	**−2.88 to −1.23**	0.52	1.38	−1.59 to 4.36	0.42	1.97	−1.73 to 5.66
G5_average_ versus G5_late_	0.55	0.76	−0.95 to 2.47	**< 0.001**	**−1.95**	**−2.68 to −1.22**	0.96	−0.27	−2.72 to 2.18	0.98	−0.27	−3.38 to 2.84
WC (cm)	All 0.67			All 0.08			All 0.48			All 0.06		
G5_early_ versus G5_average_	0.75	0.49	−1.12 to 2.11	0.12	2.57	−0.47 to 5.62	0.48	1.75	−1.83 to 5.33	0.05	4.48	−0.02 to 8.89
G5_early_ versus G5_late_	0.67	0.65	−1.13 to 2.43	0.96	0.39	−2.97 to 3.75	0.55	1.71	−2.18 to 5.61	0.54	2.21	−2.74 to 7.15
G5_average_ versus G5_late_	0.97	0.15	−1.38 to 1.69	0.18	−2.18	−5.09 to 0.73	0.99	−0.04	−3.24 to 3.17	0.40	−2.28	−6.44 to 1.88
Systolic BP (mmHg)	All 0.13			**All 0.01**			All 0.09			All 0.08		
G5_early_ versus G5_average_	0.13	2.45	−0.54 to 5.48	0.06	4.52	−0.08 to 9.12	0.43	2.77	−2.50 to 8.05	0.52	2.25	−2.64 to 7.14
G5_early_ versus G5_late_	0.22	2.37	−0.96 to 5.69	0.94	−0.75	−5.85 to 4.34	0.81	−1.49	−7.23 to 4.24	0.65	−2.02	−7.40 to 3.35
G5_average_ versus G5_late_	0.99	−0.10	−2.96 to 2.76	**0.01**	**−5.27**	**−9.67 to −0.88**	0.09	−4.27	−8.99 to 0.45	0.07	−4.27	−8.80 to 0.25
Diastolic BP (mmHg)	All 0.38			**All 0.02**			All 0.18			All 0.12		
G5_early_ versus G5_average_	0.74	0.84	−1.85 to 3.54	0.51	1.25	−1.40 to 3.90	0.29	2.23	−1.27 to 5.74	0.98	−0.30	−4.17 to 3.57
G5_early_ versus G5_late_	0.87	−0.64	−3.61 to 2.33	0.37	−1.69	−4.62 to 1.25	0.99	0.20	−3.62 to 4.01	0.19	−3.13	−7.38 to 1.13
G5_average_ versus G5_late_	0.36	−1.49	−1.85 to 3.54	**0.02**	**−2.94**	**−5.47 to −0.41**	0.28	−2.04	−5.17 to 1.10	0.15	−2.83	−6.41 to 0.75
TC (mmol/L)	All 0.12			**All 0.046**			All 0.18			All 0.31		
G5_early_ versus G5_average_	0.99	−0.01	−0.27 to 0.25	0.92	0.04	−0.22 to 0.30	0.85	−0.07	−0.39 to 0.24	0.98	0.04	−0.40 to 0.48
G5_early_ versus G5_late_	0.20	−0.21	−0.49 to 0.08	0.19	−0.21	−0.50 to 0.07	0.20	−0.24	−0.58 to 0.09	0.54	−0.22	−0.70 to 0.27
G5_average_ versus G5_late_	0.14	−0.20	−0.45 to 0.05	**0.04**	**−0.25**	**−0.50 to −0.01**	0.31	−0.17	−0.45 to 0.11	0.30	−0.26	−0.67 to 0.15
HDL‐C (mmol/L)	All 0.39			All 0.57			All 0.13			All 0.20		
G5_early_ versus G5_average_	0.68	−0.04	−0.14 to 0.07	0.77	−0.02	−0.11 to 0.06	0.11	−0.09	−0.20 to 0.02	0.44	−0.07	−0.22 to 0.07
G5_early_ versus G5_late_	0.36	−0.07	−0.18 to 0.05	0.55	−0.04	−0.14 to 0.05	0.30	−0.08	−0.20 to 0.04	0.18	−0.12	−0.28 to 0.04
G5_average_ versus G5_late_	0.76	−0.03	−0.13 to 0.07	0.87	−0.02	−0.10 to 0.06	0.90	0.02	−0.08 to 0.12	0.70	−0.05	−0.18 to 0.09
LDL‐C (mmol/L)	All 0.20			All 0.17			All 0.36			All 0.70		
G5_early_ versus G5_average_	0.99	0.01	−0.22 to 0.23	0.65	0.08	−0.14 to 0.30	0.99	0.02	−0.26 to 0.30	0.98	0.03	−0.36 to 0.42
G5_early_ versus G5_late_	0.33	−0.15	−0.39 to 0.10	0.69	−0.08	−0.33 to 0.16	0.57	−0.13	−0.44 to 0.18	0.85	−0.10	−0.53 to 0.33
G5_average_ versus G5_late_	0.20	−0.16	−0.37 to 0.06	0.15	−0.17	−0.38 to 0.04	0.35	−0.15	−0.40 to 0.11	0.68	−0.13	−0.49 to 0.23
TG^b^ (mmol/L)	All 0.82			**All 0.01**			All 0.49			All 0.20		
G5_early_ versus G5_average_	0.87	0.03	−0.12 to 0.18	0.34	−0.10	−0.27 to 0.07	0.99	0.01	−0.19 to 0.21	0.43	0.11	−0.10 to 0.33
G5_early_ versus G5_late_	1.0	−0.0004	−0.17 to 0.17	**0.01**	**−0.24**	**−0.43 to −0.06**	0.67	−0.08	−0.30 to 0.14	0.96	−0.03	−0.27 to 0.21
G5_average_ versus G5_late_	0.86	−0.03	−0.18 to 0.11	0.09	−0.14	−0.30 to 0.02	0.48	−0.09	−0.27 to 0.09	0.22	−0.14	−0.34 to 0.06
Glucose (mmol/L)	—			All 0.24			All 0.36			All 0.28		
G5_early_ versus G5_average_	—	—	—	0.96	0.02	−0.14 to 0.18	0.41	−0.09	−0.25 to 0.07	0.61	0.10	−0.15 to 0.34
G5_early_ versus G5_late_	—	—	—	0.52	−0.08	−0.26 to 0.09	0.39	−0.10	−0.28 to 0.08	0.90	−0.05	−0.32 to 0.22
G5_average_ versus G5_late_	—	—	—	0.22	−0.10	−0.25 to 0.04	0.98	−0.01	−0.16 to 0.14	0.28	−0.15	−0.37 to 0.08
Insulin^b^ (mU/L)	—			All 0.68			All 0.88			**All 0.04**		
G5_early_ versus G5_average_	—	—	—	0.99	0.001	−0.18 to 0.18	0.88	0.04	−0.17 to 0.26	0.06	0.26	−0.01 to 0.53
G5_early_ versus G5_late_	—	—	—	0.78	−0.06	−0.26 to 0.14	0.98	0.02	−0.22 to 0.25	0.89	0.06	−0.24 to 0.35
G5_average_ versus G5_late_	—	—	—	0.68	−0.06	−0.22 to 0.11	0.95	−0.03	−0.22 to 0.17	0.13	−0.20	−0.45 to 0.04
HOMA‐IR^b^	—			All 0.53			All 0.68			**All 0.03**		
G5_early_ versus G5_average_	—	—	—	0.99	0.01	−0.19 to 0.20	0.79	0.06	−0.16 to 0.28	0.05	0.28	−0.003 to 0.56
G5_early_ versus G5_late_	—	—	—	0.68	−0.07	−0.29 to 0.14	0.99	−0.01	−0.24 to 0.23	0.92	0.05	−0.26 to 0.36
G5_average_ versus G5_late_	—	—	—	0.53	−0.08	−0.25 to 0.09	0.72	−0.07	−0.26 to 0.13	0.10	−0.23	−0.49 to 0.03
CRP*yyy (mg/L)	—			—			All 0.91			**All 0.01**		
G5_early_ versus G5_average_	—	—	—	—	—	—	0.99	0.004	−0.52 to 0.53	**0.04**	**0.55**	**0.02 to 1.08**
G5_early_ versus G5_late_	—	—	—	—	—	—	0.94	−0.08	−0.65 to 0.49	0.99	−0.03	−0.62 to 0.55
G5_average_ versus G5_late_	—	—	—	—	—	—	0.91	−0.08	−0.56 to 0.39	**0.02**	**−0.58**	**−1.07 to −0.09**
GlycA (μmol/L)	—			—			—			**All 0.01**		
G5_early_ versus G5_average_	—	—	—	—	—	—	—	—	—	**0.02**	**0.10**	**0.01 to 0.18**
G5_early_ versus G5_late_	—	—	—	—	—	—	—	—	—	0.92	0.02	−0.08 to 0.11
G5_average_ versus G5_late_	—	—	—	—	—	—	—	—	—	**0.03**	**−0.08**	**−0.16 to −0.01**

*Note:* **Bolded** = Statistically significant.

^a^ Difference in means between X and Y, comparing to X.

^b^ Logarithm converted.

## 4 Discussion

Cardiometabolic measures in adolescence and young adulthood have been found to correspond to cardiovascular disease risk and measures later in life [26]. We found differences in cardiometabolic measures between the groups of earlier versus later pubertal development, even within normal ranges. In females, earlier pubertal development was associated with higher BMI and WC, both before and at the onset of puberty, and in young adulthood. In males, our findings indicate that earlier pubertal development may be disadvantageous for glucose metabolism because of high insulin and insulin resistance observed at 26 years of age.

### 4.1 BMI and WC and Height

In line with previous studies [12], we found that earlier menarche and onset of pubertal development in females are associated with higher BMI and WC at the beginning of breast development and in young adulthood. Our findings would indicate a clinically relevant difference between the earliest and latest groups for B2 and M in young adulthood 2.5–3.1 kg/m^2^ in BMI and 5.4–6.4 cm in WC. We found no difference in BMI or WC between the groups at the age of menarche, as menarche is most strongly hormonally regulated [27]. Low BMI may also be the cause of later pubertal onset/menarche as seen in eating disorders or athletes [28, 29]. It seems those with earlier (early < average < late) onset of puberty and earlier menarche are shorter at the pubertal milestone in question. However, they are taller before puberty, possibly reflecting accelerated pre‐adolescent growth. Although insignificant, females in the M_early_ group were taller by age compared to the other groups; they ended up shorter by average than M_average_ and M_late_ (Figure 3).

Regarding males there have been contradicting findings on how weight may affect pubertal development: it has been postulated that overweight may cause earlier puberty and obesity a delay. In our data there were only 5 boys who were classified as obese at the G2 point and there were no significant differences in results whether they were included in the analyses or not. Thus, they were included in all analyses. Those with earlier (early < average < late) onset and end of testicular growth were shorter at the pubertal milestone in question. However, before puberty at 7 years of age, the G2early and G5early groups were taller than G2late and G5late groups. Additionally, at the G2 age both BMI and WC were smaller in the earlier group(s) compared to the latest group. As seen in Figures 1, 2, 3 the mean curve of these measures was higher for earlier puberty (early > average > late). Thus, this may indicate smaller stature because of age rather than a cause of earlier puberty.

Higher BMI and WC in adolescence and young adulthood were associated with higher BMI later in adulthood, which may increase the risk of associated diseases later in life [26].

### 4.2 Blood Pressure

BP in children starts to increase when the children enter puberty and reaches adult levels by the end of puberty [9]. Earlier puberty has been found to be associated with higher BP later in life [14]. In our study, earlier breast development was associated with higher diastolic BP at 20 years of age and with higher systolic and diastolic BP at 26 years of age, suggesting that differences in BP increase later in adulthood.

Both systolic and diastolic BP seemed to be lower in the G5_average_ group compared to the G5_late_ group, but these differences did not persist into adulthood. Our findings do not seem to indicate that earlier puberty in males associates with BP later in life.

### 4.3 Serum Lipids

Previous studies on the association between pubertal development and adult lipid values have been contradicting [12, 15]. We found no association between pubertal timing and blood lipids in young adulthood in either females or males. At the B2, G2, and G5 milestones, there seemed to be some differences in blood lipids between the groups, but these did not persist to adulthood.

### 4.4 Glucose Metabolism

There were no differences in fasting glucose levels at any measuring point between any groups for females or males.

In females the M_average_ group seemed to have higher insulin and HOMA‐IR levels than M_late_ at 20 years of age, but these differences were not persistent at 26 years of age. In our study, contrary to some previous findings, earlier puberty was not associated with insulin resistance in young adulthood [13].

In contrast, in males both earlier beginning and earlier end of genital growth seemed to be associated with higher insulin and HOMA‐IR levels at 26 years of age. This suggests that earlier puberty may increase insulin resistance later in adulthood and thereby presents a risk factor for type 2 diabetes.

### 4.5 Inflammatory Biomarkers

We found no difference between groups in CRP or GlycA levels at any measuring point for females. Use of oral contraceptives has been associated with higher CRP concentrations than in non‐users [30]. There were no vast differences between groups in use of oral contraception (Table S5). Among 26‐year‐old males, G5_average_ had lower levels of CRP than both G5_early_ and G5_late_, which seemed to have similar levels. This contradicts a previous study that found CRP levels to differ by pubertal stage for females but not for males [17]. However, another study found no association between pubertal development and CRP‐levels [31]. Higher concentrations of GlycA associate with adverse cardiometabolic measures [18]. At 26 years of age, G5_average_ had higher GlycA than G5_early_ and G5_late_.

The association between pubertal development and inflammatory markers predicting cardiometabolic risk remains unclear.

### 4.6 Strengths and Limitations

The prospective setting of the study, a large number of frequently and repeatedly studied participants, and the well‐established and meticulously applied methods were obvious strengths of the study. Making it possible to study the effect of puberty both during pubertal development and in adulthood in the same study population. An additional strength was the steady follow‐up of pubertal development that was clinically evaluated with proper measurements and not self‐reported. In contrast to self‐assessment of Tanner B staging in particular, that is error prone and often overestimated in overweight females [32]. Most participants were appropriate for gestational age at birth and remained normal weight throughout the study period with pubertal development within normal range, enabling us to investigate the impact of pubertal development in a ‘normally’ growing population.

Potential limitations include the loss to follow‐up that is inevitable in every longitudinal study. We have previously shown that there were no significant differences between those who continued in the study and those lost to follow‐up [19]. Accordingly, there should be no systemic selection bias [19]. The intervention group had study visits twice a year and the control group only once a year. This is why we chose to include only the annual pubertal assessments. The intervention group had a higher probability of meeting the intervention targets of quality of dietary fat and fibre intake [21]. We have previously shown that participants who achieved more dietary targets had lower BP, LDL‐C, fasting glucose and insulin, and HOMA‐IR [21, 33]. However, because of the limited number of participants and as the distribution of intervention versus control was quite equal, we did not differentiate study groups in this study. Pubertal development was assessed from the age of 9 years onward, which may be a limitation as normal pubertal development for females may already begin at 8 years of age. The beginning of menstruation was self‐reported by most with a monthly accuracy on annual visits. Possible inaccuracies in reporting the age at menarche were accounted for by excluding the 26 females whose pubertal duration came out to be < 0 year. The 3 males who seemed to have a pubertal duration < 1 year due to the annual assessment were also excluded. The final sample size of 278 females and 235 males is quite small and a potential limitation to the interpretation of results. Information on parents' pubertal development was not available. Lastly, all participants in the STRIP study were Caucasian and thus the results may not be generalizable to other ethnicities.

## 5 Conclusions

Early pubertal development in females was associated with higher BMI, WC, and BP in young adulthood. We found earlier breast development to associate with higher BMI from puberty onward with increasing differences. Early pubertal development in males was associated with increased insulin levels and insulin resistance at adult age. The incidence of childhood overweight is on the rise and may be contributing to earlier pubertal timing in females. Thus, decreasing childhood overweight and obesity could come with numerous public health benefits. Additionally, more studies, especially at higher ages, are needed to study if pubertal timing further affects BP, blood lipids, or glucose metabolism.

## Author Contributions

**Katja Pahkala:** conceptualization, investigation, writing – review and editing. **Olli Raitakari:** conceptualization, investigation, writing – review and editing. **Jorma Viikari:** conceptualization, investigation, writing – review and editing. **Saga Nummela:** writing – original draft, conceptualization, formal analysis. **Jorma Toppari:** conceptualization, writing – review and editing. **Harri Niinikoski:** conceptualization, investigation, writing – review and editing. **Tapani Rönnemaa:** conceptualization, investigation, writing – review and editing. **Sinikka Karppinen:** conceptualization, writing – review and editing.

## Funding

This research was funded by the Academy of Finland (Grants 206374, 294834, 251360, 275595, 307996, and 322112), the Juho Vainio Foundation, the Finnish Foundation for Cardiovascular Research, the Finnish Ministry of Education and Culture, the Finnish Cultural Foundation, the Sigrid Jusélius Foundation, Special Governmental grants for Health Sciences Research (Turku University Hospital), the Yrjö Jahnsson Foundation, the Finnish Medical Foundation, the Turku University Foundation, and the John Pelander foundation (Grant 082106).

## Conflicts of Interest

The authors declare no conflicts of interest.

## Supporting information


**Figure S1:** Visual presentation of mean total cholesterol (TC) by group. (A) Beginning of breast development (B2) early, average, late (B) Beginning of testicular development (G2) early, average, late (C) Menarche (M) early, average, late (D) End of testicular development (G5) early, average, late.

**Figure S2:** Visual presentation of mean high density lipoprotein cholesterol (HDL‐C) by group. (A) Beginning of breast development (B2) early, average, late (B) Beginning of testicular development (G2) early, average, late (C) Menarche (M) early, average, late (D) End of testicular development (G5) early, average, late.

**Figure S3:** Visual presentation of mean low density lipoprotein cholesterol (LDL‐C) by group. (A) Beginning of breast development (B2) early, average, late (B) Beginning of testicular development (G2) early, average, late (C) Menarche (M) early, average, late (D) End of testicular development (G5) early, average, late.

**Figure S4:** Visual presentation of mean triglycerides (TG) by group. (A) Beginning of breast development (B2) early, average, late (B) Beginning of testicular development (G2) early, average, late (C) Menarche (M) early, average, late (D) End of testicular development (G5) early, average, late.

**Figure S5:** Visual presentation of mean HOMA‐IR by group. (A) Beginning of breast development (B2) early, average, late (B) Beginning of testicular development (G2) early, average, late (C) Menarche (M) early, average, late (D) End of testicular development (G5) early, average, late.

**Table S1:** Descriptive values by group of beginning of breast development (B2).

**Table S2:** Descriptive values by group of menarche (M).

**Table S3:** Descriptive values by group of beginning of testicular development (G2).

**Table S4:** Descriptive values by group of end of testicular development (G5).

**Table S5:** Distribution of intervention and control group in groups of pubertal timing.

## Data Availability

Research data are not shared.
