# Supplementary material for: Pubertal Timing Associates With Cardiometabolic Markers During Puberty and in Young Adulthood
Source: Acta Paediatr. 2026 May 29;115(10):2168–90. doi: 10.1111/apa.70624 (PMC13552584; doi:10.1111/apa.70624)
Supplement: Supplementary file 1 [file APA-115-2168-s001.docx]

Table S4. Descriptive values by group of end of testicular development (G5)

|  | G5_early_ N= 58 | | | G5_average_ N= 111 | | | G5_late_ N= 67 | | |
| --- | --- | --- | --- | --- | --- | --- | --- | --- | --- |
| Age at G5 | 13–15 years, 24.9 % | | | 16 years, 47.4 % | | | 17–20 years, 27.6 % | | |
|  | Mean  (SD) | Range | n | Mean  (SD) | Range | n | Mean  (SD) | Range | n |
| At 7 years of age | | | | | | | | | |
| BMI  (kg/m^2^) | 16.0  (1.4) | 13.6–19.9 | 56 | 15.7  (1.5) | 12.9–20.6 | 108 | 15.6  (1.8) | 12.1–20.4 | 66 |
| **Height**  **(cm)** | **126.7**  **(4.6)** | **117.0–134.3** | **56** | **124.9**  **(4.5)** | **115.6–137.3** | **108** | **124.1**  **(4.8)** | **112.8–133.8** | **66** |
| WC  (cm) | 55.7  (3.4) | 50.0–65.0 | 52 | 55.2  (4.1) | 47.0–67.0 | 98 | 55.0  (4.3) | 47.0–67.5 | 61 |
| sBP  (mmHg) | 101.4  (8.8) | 81.0–127.0 | 56 | 98.9  (7.4) | 76.0–121.0 | 108 | 99.0  (7.3) | 80.0–116.0 | 66 |
| dBP  (mmHg) | 58.5  (6.6) | 43.0–75.0 | 56 | 57.6  (7.4) | 40.0–93.0 | 108 | 59.1  (6.4) | 42.0–74.0 | 66 |
| TC  (mmol/L) | 4.4  (0.6) | 2.6–5.7 | 56 | 4.4  (0.7) | 3.0–6.4 | 104 | 4.6  (0.7) | 3.2–7.2 | 64 |
| HDL-C  (mmol/L) | 1.3  (0.2) | 0.8–1.7 | 56 | 1.3  (0.3) | 0.7–2.5 | 104 | 1.4  (0.3) | 0.7–2.1 | 64 |
| LDL-C  (mmol/L) | 2.8  (0.6) | 1.1–3.9 | 56 | 2.8  (0.6) | 1.7–4.2 | 104 | 2.9  (0.6) | 1.8–5.2 | 64 |
| TG*  (mmol/L) | 0.7  (0.5–0.8) | 0.2–2.1 | 56 | 0.6  (0.5–0.8) | 0.3–1.2 | 104 | 0.6  (0.5–0.8) | 0.3–2.0 | 64 |
| At G5 | | | | | | | | | |
| BMI  (kg/m^2^) | 21.2 (3.9) | 15.7–37.4 | 58 | 20.6 (2.7) | 15.6–30.7 | 111 | 21.2 (3.5) | 15.8–35.0 | 67 |
| **Height**  **(cm)** | **177.2**  **(7.6)** | **160.5–193.5** | **58** | **177.3**  **(6.5)** | **164.8–192.0** | **111** | **179.2**  **(6.7)** | **165.9–194.6** | **67** |
| WC  (cm) | 77.1 (9.4) | 66.0–110.0 | 58 | 74.5 (6.7) | 63.0–103.0 | 109 | 76.7 (8.4) | 64.5–104.0 | 67 |
| **sBP**  **(mmHg)** | **122.8** (**11.6**) | **98.7–151.7** | **58** | **118.2** (**11.5**) | **94.7–145.3** | **111** | **123.5** (**13.2**) | **99.0–160.0** | **67** |
| **dBP**  **(mmHg)** | **61.0** (**7.5**) | **46.5–75.5** | **58** | **59.7** (**6.4**) | **46.5–79.5** | **111** | **62.7** (**7.3**) | **48.0–79.5** | **67** |
| **TC**  **(mmol/L)** | **3.8** (**0.7**) | **2.0–5.3** | **58** | **3.7** (**0.7**) | **2.4–6.1** | **110** | **4.0** (**0.7**) | **2.7–5.5** | **67** |
| HDL-C  (mmol/L) | 1.0 (0.2) | 0.6–1.5 | 58 | 1.1 (0.2) | 0.56–2.0 | 109 | 1.1 (0.2) | 0.7–1.8 | 67 |
| LDL-C  (mmol/L) | 2.3 (0.6) | 0.8–3.8 | 58 | 2.2 (0.6) | 1.0–4.1 | 109 | 2.4 (0.6) | 1.1–2.4 | 67 |
| **TG***  **(mmol/L)** | **0.8** (**0.6–1.0**) | **0.2–3.5** | **58** | **0.8** (**0.7–1.1**) | **0.4–2.5** | **110** | **0.9** (**0.7–1.9**) | **0.56–2.7** | **67** |
| Glucose  (mmol/L) | 5.0 (0.4) | 4.0–5.8 | 48 | 5.0 (0.4) | 2.9–5.9 | 110 | 5.1 (0.4) | 4.0–6.5 | 67 |
| Insulin*  (mU/L) | 7.8 (6.7–9.7) | 2.5–24.5 | 48 | 7.9 (6.1–9.7) | 3.4–51.0 | 109 | 7.6 (5.9–11.1) | 3.2–35.7 | 65 |
| HOMA-IR* | 1.7 (1.4–2.2) | 0.5–5.5 | 48 | 1.7 (1.4–2.2) | 0.6–12.7 | 109 | 1.8 (1.3–2.5) | 0.6–8.7 | 65 |
| At 20 years of age | | | | | | | | | |
| BMI  (kg/m^2^) | 23.5 (4.7) | 17.5–42.3 | 42 | 22.8 (2.8) | 15.3–31.7 | 93 | 22.5 (3.1) | 16.8–31.6 | 59 |
| Height  (cm) | 182.5  (5.8) | 169.4–193.9 | 42 | 180.8  (6.0) | 165.5–192.9 | 94 | 181.1  (6.9) | 166.6–195.9 | 59 |
| WC  (cm) | 81.9 (10.3) | 67.5–125.0 | 42 | 80.1 (7.2) | 63.0–105.0 | 94 | 80.1 (8.0) | 64.0–106.0 | 59 |
| sBP  (mmHg) | 127.4 (11.0) | 100.7–153.3 | 42 | 124.6 (12.6) | 92.7–158.7 | 94 | 128.9 (11.8) | 101.5–160.3 | 59 |
| dBP  (mmHg) | 66.7 (7.8) | 49.0–84.0 | 42 | 64.5 (7.9) | 49.5–89.0 | 94 | 66.5 (8.3) | 47.0–86.5 | 59 |
| TC  (mmol/L) | 4.0 (0.7) | 2.0–5.1 | 42 | 4.0 (0.7) | 2.0–5.8 | 92 | 4.2 (0.7) | 2.7–6.3 | 58 |
| HDL-C  (mmol/L) | 1.1 (0.2) | 0.6–1.6 | 42 | 1.2 (0.3) | 0.5–2.0 | 92 | 1.2 (0.2) | 0.7–1.8 | 58 |
| LDL-C  (mmol/L) | 2.4 (0.6) | 0.6–3.8 | 42 | 2.4 (0.7) | 0.8–3.7 | 92 | 2.5 (0.6) | 1.3–4.9 | 58 |
| TG*  (mmol/L) | 0.9 (0.7–1.3) | 0.2–2.3 | 42 | 0.9 (0.7–1.1) | 0.3–3.3 | 92 | 1.0 (0.7–1.4) | 0.4–2.7 | 58 |
| Glucose  (mmol/L) | 4.9 (0.4) | 4.2–5.8 | 42 | 4.9 (0.4) | 4.2–5.9 | 92 | 5.0 (0.4) | 4.3–6.1 | 58 |
| Insulin*  (mU/L) | 6.4 (5.3–8.1) | 1.7–26.8 | 42 | 5.9 (4.7–8.7) | 1.6–46.4 | 94 | 6.4 (4.9–8.4) | 1.9–21.5 | 57 |
| HOMA-IR* | 1.4 (1.0–1.8) | 0.4–6.2 | 42 | 1.3 (1.0–1.8) | 0.3–4.6 | 92 | 1.4 (1.1–1.8) | 0.4–4.5 | 57 |
| CRP*  (mg/L) | 0.6 (0.3–1.4) | 0.1–42.4 | 42 | 0.8 (0.3–1.5) | 0.1–10.9 | 92 | 0.9 (0.3–1.7) | 0.1–46.3 | 58 |
| At 26 years of age | | | | | | | | | |
| BMI  (kg/m^2^) | 25.6 (3.6) | 18.4–36.2 | 33 | 24.6 (3.5) | 18.8–35.1 | 69 | 24.8 (3.8) | 17.2–33.9 | 42 |
| Height  (cm) | 183.2  (6.1) | 170.6–194.3 | 33 | 181.0  (6.8) | 166.6–193.7 | 69 | 181.3  (7.0) | 169.5–196.7 | 42 |
| WC  (cm) | 89.1 (9.2) | 74.2–114.9 | 33 | 84.6 (8.3) | 71.1–110.9 | 69 | 86.9 (9.9) | 70.2–108.3 | 42 |
| sBP  (mmHg) | 127.5 (9.5) | 109.3–148.0 | 33 | 125.3 (9.3) | 94.3–149.3 | 69 | 129.6 (10.7) | 102.3–148.0 | 42 |
| dBP  (mmHg) | 73.2 (8.2) | 56.3–93.3 | 33 | 73.5 (7.4) | 53.0–95.0 | 69 | 76.4 (7.8) | 64.0–103.3 | 42 |
| TC  (mmol/L) | 4.5 (0.7) | 2.8–5.7 | 33 | 4.4 (0.9) | 2.1–7.1 | 69 | 4.7 (1.0) | 2.7–7.8 | 42 |
| HDL-C  (mmol/L) | 1.1 (0.3) | 0.6–1.8 | 33 | 1.2 (0.3) | 0.5–1.9 | 69 | 1.2 (0.3) | 0.7–1.8 | 42 |
| LDL-C  (mmol/L) | 2.8 (0.7) | 1.0–4.0 | 33 | 2.8 (0.8) | 0.6–5.2 | 69 | 2.9 (0.9) | 1.0–5.7 | 42 |
| TG*  (mmol/L) | 0.9 (0.8–1.3) | 0.4–3.5 | 33 | 0.9 (0.7–1.2) | 0.4–2.2 | 69 | 1.1 (0.8–1.3) | 0.4–3.0 | 42 |
| Glucose  (mmol/L) | 5.2 (0.5) | 4.5–6.9 | 33 | 5.1 (0.4) | 4.1–6.0 | 69 | 5.2 (0.6) | 4.0–6.4 | 42 |
| **Insulin***  **(mU/L)** | **7.8** (**5.3–10.6**) | **3.2–44.4** | **33** | **6.2** (**4.4–8.2**) | **2.1–19.8** | **69** | **6.9** (**5.1–11.8**) | **1.8–77.4** | **42** |
| **HOMA-IR*** | **1.6** (**1.2–2.5**) | **0.7–13.6** | **33** | **1.4** (**1.0–1.9**) | **0.5–4.0** | **69** | **1.7** (**1.2–2.5**) | **0.4–14.1** | **42** |
| **CRP***  **(mg/L)** | **0.9** (**0.5–1.9**) | **0.1–9.5** | **33** | **0.4** (**0.3–1.0**) | **0.1–6.7** | **69** | **0.9** (**0.4–2.2**) | **0.1–32.4** | **42** |
| **GlycA**  **(μmol/L)** | **1.4** (**0.2**) | **1.1–2.1** | **33** | **1.3** (**0.2**) | **1.0–1.7** | **69** | **1.4** (**0.2**) | **1.1–1.8** | **42** |

* Logarithm converted, median instead of mean, and IQR instead of SD
**Bolded** = Statistically significant differences between groups in ANOVA analyses
