# Supplementary material for: Pubertal Timing Associates With Cardiometabolic Markers During Puberty and in Young Adulthood
Source: Acta Paediatr. 2026 May 29;115(10):2168–90. doi: 10.1111/apa.70624 (PMC13552584; doi:10.1111/apa.70624)
Supplement: Supplementary file 2 [file APA-115-2168-s002.docx]

|  | Intervention group | | Control group | |
| --- | --- | --- | --- | --- |
|  | % | n | % | n |
| B2_early_ | 47 | 48 | 53 | 55 |
| B2_average_ | 41 | 31 | 59 | 76 |
| B2_late_ | 49 | 49 | 50 | 50 |
| M_early_ | 48 | 44 | 52 | 47 |
| M_average_ | 43 | 40 | 57 | 53 |
| M_late_ | 48 | 41 | 52 | 45 |
| G2_early_ | 45 | 21 | 55 | 47 |
| G2_average_ | 50 | 52 | 50 | 53 |
| G2_late_ | 51 | 42 | 49 | 41 |
| G5_early_ | 60 | 35 | 40 | 23 |
| G5_average_ | 50 | 55 | 50 | 56 |
| G5_late_ | 39 | 26 | 61 | 41 |

Table S5. Distribution of intervention and control group in groups of pubertal timing.
