# Supplementary material for: Pubertal Timing Associates With Cardiometabolic Markers During Puberty and in Young Adulthood
Source: Acta Paediatr. 2026 May 29;115(10):2168–90. doi: 10.1111/apa.70624 (PMC13552584; doi:10.1111/apa.70624)
Supplement: Supplementary file 3 [file APA-115-2168-s003.docx]

Table S1. Descriptive values by group of beginning of breast development (B2)

|  | B2_early_  N= 103 | | | B2_average_  N=76 | | | B2_late_  N=99 | | |
| --- | --- | --- | --- | --- | --- | --- | --- | --- | --- |
| Age at B2 | 8–10 years, 37.2 % | | | 11 years, 26.8 % | | | 12–16 years, 36.0 % | | |
|  | Mean  (SD) | Range | n | Mean  (SD) | Range | n | Mean  (SD) | Range | n |
| At 7 years of age | | | | | | | | | |
| **BMI**  **(kg/m^2^)** | **16.8**  **(2.2)** | **12.9–24.9** | **100** | **16.3**  **(2.2)** | **12.8–26.7** | **75** | **15.1**  **(1.5)** | **12.8–23.1** | **94** |
| **Height**  **(cm)** | **126.6**  **(5.0)** | **114.8–143.3** | **100** | **124.2**  **(4.9)** | **113.8–134.7** | **75** | **122.3**  **(4.3)** | **110.2–132.8** | **94** |
| **WC**  **(cm)** | **56.8**  **(4.5)** | **49.0–70.0** | **91** | **55.8**  **(5.7)** | **48.0–75.0** | **62** | **52.4**  **(4.0)** | **45.0–73.0** | **88** |
| **sBP**  **(mmHg)** | **103.2**  **(10.6)** | **79.0–137.0** | **100** | **98.9**  **(8.4)** | **81.0–117.0** | **75** | **100.6**  **(8.7)** | **80.0–131.0** | **93** |
| dBP  (mmHg) | 59.0  (5.9) | 45.0–74.0 | 100 | 58.8  (6.1) | 45.0–75.0 | 75 | 58.8  (7.0) | 45.0–74.0 | 93 |
| TC  (mmol/L) | 4.7  (0.7) | 3.3–7.2 | 97 | 4.6  (0.7) | 3.2–6.8 | 74 | 4.7  (0.8) | 2.9–7.3 | 90 |
| HDL-C  (mmol/L) | 1.3  (0.2) | 0.8–1.7 | 97 | 1.3  (0.2) | 0.7–1.9 | 74 | 1.3  (0.2) | 0.9–1.9 | 90 |
| LDL-C  (mmol/L) | 3.1  (0.7) | 1.8–5.7 | 97 | 3.0  (0.6) | 1.6–4.6 | 74 | 3.1  (0.7) | 1.5–5.4 | 90 |
| TG*  (mmol/L) | 0.7  (0.5–0.8) | 0.3–1.7 | 97 | 0.7  (0.5–0.8) | 0.3–1.6 | 74 | 0.7  (0.5–0.8) | 0.4–2.1 | 90 |
| At B2 | | | | | | | | | |
| **BMI**  **(kg/m^2^)** | **18.6**  **(2.8)** | **13.1–25.8** | **103** | **18.8**  **(3.3)** | **13.7–30.5** | **76** | **17.3**  (**2.1**) | **13.9–29.1** | **98** |
| **Height**  **(cm)** | **142.6**  **(5.6)** | **127.5–160.0** | **103** | **148.3**  **(6.0)** | **131.1–159.5** | **76** | **153.3**  **(6.2)** | **137.7–168.1** | **99** |
| WC  (cm) | 63.7  (7.0) | 51.0–88.0 | 97 | 65.1  (8.3) | 53.5–90.0 | 76 | 63.1  (5.9) | 52.5–92.5 | 96 |
| sBP  (mmHg) | 106.4  (9.7) | 82.0–134.7 | 100 | 106.1  (8.7) | 84.0–125.7 | 76 | 106.1  (9.5) | 90.3–138.7 | 98 |
| dBP  (mmHg) | 59.4  (6.4) | 45–72.5 | 100 | 58.2  (5.4) | 47.5–73.5 | 76 | 58.2  (6.8) | 45.5–80.5 | 98 |
| **TC**  **(mmol/L)** | **4.7**  (**0.8)** | **3.01–8.2** | **93** | **4.5**  **(0.7)** | **2.9–6.4** | **74** | **4.4**  (**0.7**) | **3.2–7.0** | **97** |
| HDL-C  (mmol/L) | 1.3  (0.2) | 0.65–1.7 | 92 | 1.3  (0.3) | 0.6–2.1 | 74 | 1.3  (0.2) | 0.67–1.9 | 97 |
| **LDL-C**  **(mmol/L)** | **3.1**  (**0.8)** | **1.3–6.4** | **91** | **2.8**  **(0.6)** | **1.7–4.3** | **74** | **2.8**  (**0.6**) | **1.6–5.3** | **97** |
| **TG***  **(mmol/L)** | **0.8**  (**0.6–1.0)** | **0.3–2.8** | **93** | **0.8**  **(0.6–1.0)** | **0.4–2.3** | **74** | **0.7**  (**0.6–0.9**) | **0.4–3.2** | **97** |
| **Glucose**  **(mmol/L)** | **4.5**  **(0.2)** | **4.3–4.8** | **10** | **4.8**  **(0.2)** | **4.6–5.0** | **18** | **4.8**  (**0.2**) | **4.6–5.0** | **7** |
| Insulin*  (mU/L) | 6.5  (4.6–8.1) | 2.3–13.8 | 24 | 7.2  (6.6–8.5) | 4.0–18.1 | 18 | 7.2  (5.9–9.1) | 5.3–12.3 | 7 |
| HOMA-IR* | 1.3  (0.9–1.5) | 0.6–1.7 | 10 | 1.6  (1.4–1.9) | 0.8–3.9 | 18 | 1.5  (1.2–1.9) | 1.1–2.8 | 7 |
| At 20 years of age | | | | | | | | | |
| **BMI**  **(kg/m^2^)** | **23.8**  (**4.3)** | **15.6–41.3** | **85** | **23.5**  **(4.8)** | **17.3–37.4** | **63** | **21.3**  (**2.8**) | **16.3–32.5** | **81** |
| **Height**  **(cm)** | 167.7  (6.5) | 151.2–184.5 | 85 | 167.6  (5.3) | 153–182.7 | 63 | 167.8  (5.8) | 154.9–180.1 | 81 |
| **WC**  **(cm)** | **76.3**  (**10.0)** | **57–114.5** | **85** | **75.6**  **(10.4)** | **63.0–115.5** | **63** | **70.7**  (**7.3**) | **59.5–101.5** | **80** |
| sBP  (mmHg) | 116.9  (11.8) | 91.3–144 | 85 | 114.5  (14.1) | 83.0–151.0 | 63 | 113.7  (11.1) | 85.5–153.0 | 81 |
| **dBP**  **(mmHg)** | **67.1**  (**7.3)** | **50.5–85** | **85** | **66.1**  **(8.8)** | **50.0–86.0** | **63** | **64.1**  (**7.1**) | **48.0–81.5** | **81** |
| TC  (mmol/L) | 4.6  (0.8) | 3.2–7.1 | 83 | 4.7  (0.9) | 3.2–7.4 | 63 | 4.6  (0.8) | 3.0–6.3 | 78 |
| HDL-C  (mmol/L) | 1.5  (0.3) | 0.9–2.8 | 83 | 1.5  (0.4) | 0.8–2.5 | 63 | 1.5  (0.3) | 1.0–2.2 | 78 |
| LDL-C  (mmol/L) | 2.6  (0.7) | 1.1–4.9 | 83 | 2.6  (0.8) | 1.2–5.1 | 63 | 2.7  (0.7) | 1.3–4.1 | 78 |
| TG*  (mmol/L) | 1.1  (0.7–1.3) | 0.5–3.1 | 83 | 1.0  (0.8–1.4) | 0.4–3.2 | 63 | 1.0  (0.7–1.3) | 0.4–2.9 | 78 |
| Glucose  (mmol/L) | 4.7  (0.4) | 4.0–6.0 | 83 | 4.8  (0.5) | 4.1–6.4 | 63 | 4.8  (0.4) | 3.7–6.8 | 78 |
| Insulin*  (mU/L) | 6.3  (4.9–8.9) | 1.8–22.5 | 82 | 6.6  (4.9–9.3) | 2.5–43.3 | 63 | 6.3  (5.0–8.4) | 3.2–21.1 | 78 |
| HOMA-IR* | 1.3  (1.0–1.8) | 0.4–5.0 | 82 | 1.4  (1.0–2.0) | 0.5–10.2 | 63 | 1.3  (1.0–1.9) | 0.7–5.7 | 78 |
| **CRP***  **(mg/L)** | **1.4**  (**0.5–2.9)** | **0.2–40.6** | **83** | **1.7**  **(0.5–4.1)** | **0.1–40.7** | **63** | **1.1**  (**0.4–2.3**) | **0.1–12.0** | **78** |
| At 26 years of age | | | | | | | | | |
| **BMI**  **(kg/m^2^)** | **24.9**  (**5.2)** | **14.2–44.3** | **73** | **24.3**  **(3.8)** | **18.1–33.2** | **62** | **22.2**  (**3.7**) | **17.5–38.5** | **71** |
| **Height**  **(cm)** | 167.8  (6.4) | 156.1–185.0 | 73 | 168.3  (5.5) | 153.7–182.9 | 62 | 167.6  (6.4) | 154.6–180.9 | 71 |
| **WC**  **(cm)** | **79.3**  (**12.0)** | **54.1–122.8** | **73** | **77.6**  **(10.0)** | **62.2–106.1** | **62** | **73.0**  (**8.5**) | **61.0–102.0** | **71** |
| **sBP**  **(mmHg)** | **117.8**  (**8.7)** | **97.0–146.3** | **73** | **114.8**  **(9.7)** | **94.3–143.0** | **62** | **113.7**  (**8.4**) | **93.3–138.3** | **71** |
| **dBP**  **(mmHg)** | **71.6**  (**6.4)** | **57.3–89.7** | **73** | **70.8**  **(7.3)** | **56.0–90.7** | **62** | **68.6**  (**6.1**) | **54.7–82.7** | **71** |
| TC  (mmol/L) | 4.5  (0.8) | 2.7–6.6 | 73 | 4.7  (1.0) | 2.8–7.2 | 62 | 4.6  (0.9) | 2.4–7.6 | 69 |
| HDL-C  (mmol/L) | 1.4  (0.3) | 0.8–2.1 | 73 | 1.5  (0.4) | 0.8–2.4 | 62 | 1.5  (0.3) | 0.7–2.5 | 69 |
| LDL-C  (mmol/L) | 2.6  (0.7) | 0.9–4.4 | 73 | 2.8  (0.7) | 1.2–4.9 | 62 | 2.8  (0.7) | 1.4–4.7 | 69 |
| TG*  (mmol/L) | 0.9  (0.6–1.3) | 0.4–2.4 | 73 | 0.9  (0.6–1.3) | 0.3–2.4 | 62 | 0.9  (0.7–1.1) | 0.4–2.3 | 69 |
| Glucose  (mmol/L) | 5.0  (0.5) | 3.2–7.0 | 73 | 5.0  (0.4) | 4.0–5.8 | 62 | 4.9  (0.5) | 4.0–6.0 | 69 |
| Insulin*  (mU/L) | 6.7  (5.3–9.1) | 3.0–43.0 | 73 | 6.6  (5.3–9.2) | 2.7–17.9 | 62 | 6.9  (5.4–8.8) | 2.8–20.0 | 69 |
| HOMA-IR* | 1.5  (1.1–2.0) | 0.6–9.6 | 73 | 1.5  (1.1–2.0) | 0.6–4.6 | 62 | 1.5  (1.1–1.9) | 0.56–5.3 | 69 |
| CRP*  (mg/L) | 1.2  (0.6–3.5) | 0.1–15.1 | 73 | 0.9  (0.4–2.5) | 0.12–32.1 | 62 | 1.0  (0.4–2.0) | 0.1–9.3 | 69 |
| GlycA  (μmol/L) | 1.3  (0.2) | 0.9–1.9 | 73 | 1.3  (0.2) | 0.8–1.9 | 62 | 1.3  (0.2) | 1.0–1.6 | 69 |

* Logarithm converted, median instead of mean, and interquartile range (IQR) instead of SD
**Bolded** = Statistically significant differences between groups in ANOVA analyses
