# Supplementary material for: Pubertal Timing Associates With Cardiometabolic Markers During Puberty and in Young Adulthood
Source: Acta Paediatr. 2026 May 29;115(10):2168–90. doi: 10.1111/apa.70624 (PMC13552584; doi:10.1111/apa.70624)
Supplement: Supplementary file 4 [file APA-115-2168-s004.docx]

Table S2. Descriptive values by group of menarche (M)

|  | M_early_ N= 91 | | | M_average_ N= 93 | | | M_late_ N= 86 | | |
| --- | --- | --- | --- | --- | --- | --- | --- | --- | --- |
| Age at menarche | 10–12.3 years, 33 % | | | 12.4–13.4 years, 33 % | | | 13.5–17 years, 33 % | | |
|  | Mean  (SD) | Range | n | Mean  (SD) | Range | n | Mean  (SD) | Range | n |
| At 7 years of age | | | | | | | | | |
| **BMI**  **(kg/m^2^)** | **16.7**  **(2.5)** | **12.9–26.7** | **90** | **16.3**  **(2.1)** | **12.8–23.2** | **91** | **15.3**  **(1.4)** | **12.8–19.7** | **88** |
| **Height**  **(cm)** | **125.7**  **(5.0)** | **114.8–143.3** | **90** | **124.6**  **(4.8)** | **113.8–136.7** | **91** | **123.1**  **(5.1)** | **110.2–137.8** | **88** |
| **WC**  **(cm)** | **56.4**  **(5.2)** | **48.5–74.0** | **85** | **55.3**  **(5.2)** | **48.0–75.0** | **77** | **53.1**  **(4.2)** | **45.0–68.0** | **79** |
| sBP  (mmHg) | 101.6  (10.7) | 79.0–137.0 | 90 | 100.3  (9.4) | 80.0–122.0 | 90 | 101.4  (8.3) | 84.0–131.0 | 88 |
| dBP  (mmHg) | 58.9  (6.2) | 45.0–75.0 | 90 | 58.2  (6.0) | 45.0–73.0 | 90 | 59.4  (6.8) | 46.0–74.0 | 88 |
| TC  (mmol/L) | 4.6  (0.7) | 3.3–6.5 | 88 | 4.8  (0.7) | 3.1–6.8 | 88 | 4.7  (0.8) | 2.9–7.3 | 85 |
| HDL-C  (mmol/L) | 1.3  (0.2) | 0.7–1.9 | 88 | 1.3  (0.2) | 0.8–1.9 | 88 | 1.3  (0.2) | 0.9–1.9 | 85 |
| LDL-C  (mmol/L) | 3.0  (0.6) | 1.6–4.5 | 88 | 3.1  (0.7) | 1.8–4.7 | 88 | 3.1  (0.7) | 1.5–5.7 | 85 |
| TG*  (mmol/L) | 0.7  (0.5–0.8) | 0.3–1.6 | 88 | 0.7  (0.5–0.8) | 0.3–2.1 | 88 | 0.7  (0.5–0.8) | 0.4–1.6 | 85 |
| At menarche | | | | | | | | | |
| BMI  (kg/m^2^) | 19.5  (3.2) | 13.4–28.6 | 91 | 19.2  (3.1) | 14.1–30.6 | 93 | 18.5  (2.1) | 14.2–28.0 | 86 |
| Height  (cm) | 154.7  (5.9) | 140.2–170.2 | 91 | 158.5  (6.9) | 145.0–174.4 | 93 | 161.5  (6.8) | 142.8–176.8 | 86 |
| WC  (cm) | 67.5  (7.5) | 54.5–88.0 | 91 | 67.8  (7.5) | 57.0–94.0 | 93 | 67.7  (6.7) | 58.0–96.0 | 86 |
| sBP  (mmHg) | 109.3  (10.3) | 86.3–146.0 | 91 | 107.2  (10.9) | 71.3–142.0 | 93 | 110.0  (9.1) | 92.3–139.0 | 85 |
| dBP  (mmHg) | 58.2  (6.2) | 46.0–73.5 | 91 | 58.4  (6.2) | 32.0–73.5 | 93 | 59.4  (6.7) | 45.5–80.5 | 85 |
| TC  (mmol/L) | 4.2  (0.6) | 2.7–6.4 | 90 | 4.3  (0.7) | 2.1–6.0 | 92 | 4.4  (0.7) | 3.0–6.4 | 86 |
| HDL-C  (mmol/L) | 1.2  (0.2) | 0.8–1.8 | 90 | 1.2  (0.3) | 0.6–1.9 | 92 | 1.2  (0.2) | 0.7–1.7 | 86 |
| LDL-C  (mmol/L) | 2.6  (0.5) | 1.1–4.0 | 89 | 2.6  (0.6) | 1.2–4.3 | 92 | 2.8  (0.6) | 1.6–4.9 | 86 |
| TG*  (mmol/L) | 0.8  (0.6–1.0) | 0.4–4.5 | 90 | 0.8  (0.6–0.9) | 0.3–3.1 | 92 | 0.8  (0.7–0.9) | 0.4–2.5 | 86 |
| Glucose  (mmol/L) | 5.0  (0.27) | 4.5–5.5 | 16 | 4.9  (0.32) | 4.5–5.3 | 11 | 4.9  (0.27) | 4.2–5.3 | 31 |
| Insulin*  (mU/L) | 7.9  (5.9–9.8) | 4.0–21.3 | 16 | 9.8  (7.9–11.0) | 5.9–44.9 | 11 | 7.2  (5.9–10.4) | 2.0–15.5 | 31 |
| HOMA-IR* | 1.8  (1.3–2.2) | 0.8–5.2 | 16 | 1.9  (1.7–2.5) | 1.2–9.7 | 11 | 1.6  (1.2–2.3) | 0.4–3.7 | 31 |
| At 20 years of age | | | | | | | | | |
| **BMI**  **(kg/m^2^)** | **24.4**  (**4.9**) | **17.1–37.4** | **82** | **22.6**  (**4.2**) | **15.6–41.3** | **73** | **21.3**  (**2.2**) | **16.3–26.3** | **74** |
| **Height**  (cm) | **166.2**  **(5.4)** | **151.2**–181.2 | **82** | **168.3**  **(5.9)** | **154.9**–181.5 | **73** | **168.7**  **(6.3)** | **153.9**–184.5 | **74** |
| **WC**  (cm) | **77.2**  (**10.9**) | **63.0–115.5** | **82** | **74.0**  (**10.2**) | **57.0–114.5** | **73** | **70.8**  (**5.5**) | **61.5–89.0** | **74** |
| sBP  (mmHg) | 116.8  (13.2) | 91.3–151.0 | 82 | 114.3  (13.1) | 83.0–153.0 | 73 | 114.1  (10.1) | 85.5–139.7 | 74 |
| **dBP**  (mmHg) | **66.7**  (**7.5**) | **50.0–83.0** | **82** | **65.3**  (**8.5**) | **48.0–86.0** | **73** | **65.2**  (**7.3**) | **52.5–85.0** | **74** |
| TC  (mmol/L) | 4.6  (0.8) | 3.2–7.4 | 79 | 4.6  (0.8) | 3.2–6.5 | 73 | 4.6  (0.8) | 3.0–6.9 | 72 |
| HDL-C  (mmol/L) | 1.5  (0.3) | 0.8–2.8 | 79 | 1.5  (0.3) | 1.0–2.5 | 73 | 1.5  (0.3) | 1.0–2.2 | 72 |
| LDL-C  (mmol/L) | 2.7  (0.7) | 1.1–4.9 | 79 | 2.6  (0.7) | 1.6–4.3 | 73 | 2.6  (0.7) | 1.2–5.1 | 72 |
| TG*  (mmol/L) | 1.0  (0.8–1.3) | 0.4–3.1 | 79 | 1.0  (0.7–1.4) | 0.4–3.2 | 73 | 1.0  (0.7–1.3) | 0.4–2.9 | 72 |
| Glucose  (mmol/L) | 4.7  (0.4) | 4.0–6.0 | 79 | 4.8  (0.4) | 4.1–6.4 | 73 | 4.7  (0.4) | 3.7–6.8 | 72 |
| **Insulin***  **(mU/L)** | **6.8**  (**5.1–9.4**) | **1.8–22.5** | **78** | **6.8**  (**5.2–9.3**) | **2.5–43.3** | **73** | **5.8**  (**4.8–7.7**) | **3.2–16.2** | **72** |
| **HOMA-IR*** | **1.4**  (**1.1–2.0**) | **0.4–5.0** | **78** | **1.5**  (**1.1–2.0**) | **0.5–10.2** | **73** | **1.2**  (**0.9–1.6**) | **0.7–3.2** | **72** |
| CRP*  (mg/L) | 1.4  (0.5–3.3) | 0.1–33.0 | 79 | 1.2  (0.4–2.7) | 0.1–40.6 | 73 | 1.1  (0.5–2.5) | 0.1–40.7 | 72 |
| At 26 years of age | | | | | | | | | |
| **BMI**  **(kg/m^2^)** | **25.1**  (**4.4**) | **17.5–36.5** | **67** | **24.2**  (**5.3**) | **14.2–44.3** | **74** | **22.0**  (**2.5**) | **17.5–30.0** | **65** |
| **Height**  (cm) | **166.5**  **(5.1)** | **156.2**–180.4 | **67** | **168.3**  **(6.1)** | **155.1**–181.5 | **74** | **168.9**  **(6.8)** | **153.7**–185.0 | **65** |
| **WC**  **(cm)** | **79.0**  (**10.0**) | **63.2–106.1** | **67** | **77.9**  (**12.8**) | **54.1–122.8** | **74** | **72.7**  (**7.0**) | **61.0–92.3** | **65** |
| sBP  (mmHg) | 116.3  (9.0) | 96.3–143.0 | 67 | 114.9  (9.8) | 93.3–146.3 | 74 | 115.4  (8.2) | 101.3–138.3 | 65 |
| dBP  (mmHg) | 71.4  (6.4) | 56.0–90.7 | 67 | 69.6  (7.3) | 54.7–89.7 | 74 | 70.1  (6.3) | 56.7–82.7 | 65 |
| TC  (mmol/L) | 4.5  (0.9) | 2.7–7.2 | 67 | 4.7  (0.8) | 2.8–6.6 | 74 | 4.6  (0.9) | 2.4–7.6 | 63 |
| HDL-C  (mmol/L) | 1.4  (0.3) | 0.8–2.4 | 67 | 1.5  (0.3) | 0.8–2.2 | 74 | 1.5  (0.3) | 0.7–2.5 | 63 |
| LDL-C  (mmol/L) | 2.7  (0.7) | 0.9–4.9 | 67 | 2.8  (0.8) | 1.2–4.6 | 74 | 2.7  (0.7) | 1.4–4.7 | 63 |
| TG*  (mmol/L) | 1.0  (0.6–1.3) | 0.4–2.4 | 67 | 0.9  (0.7–1.3) | 0.3–2.4 | 74 | 0.8  (0.7–1.1) | 0.4–2.2 | 63 |
| Glucose  (mmol/L) | 4.9  (0.5) | 3.7–7.0 | 67 | 4.9  (0.5) | 3.2–6.2 | 74 | 5.0  (0.5) | 4.0–6.0 | 63 |
| Insulin*  (mU/L) | 6.6  (5.5–9.5) | 2.7–31.1 | 67 | 6.8  (5.1–9.2) | 3.3–43.0 | 74 | 6.6  (5.4–8.4) | 2.8–15.0 | 63 |
| HOMA-IR* | 1.5  (1.1–2.1) | 0.6–6.4 | 67 | 1.5  (1.0–2.1) | 0.7–9.6 | 74 | 1.5  (1.1–1.9) | 0.6–3.9 | 63 |
| CRP*  (mg/L) | 1.2  (0.6–2.7) | 0.1–15.6 | 67 | 1.0  (0.4–2.7) | 0.1–32.1 | 74 | 1.1  (0.5–2.0) | 0.1–13.7 | 63 |
| GlycA  (μmol/L) | 1.3  (0.2) | 0.9–1.9 | 67 | 1.3  (0.2) | 0.8–1.9 | 74 | 1.3  (0.1) | 1.0–1.6 | 63 |

* Logarithm converted, median instead of mean, and IQR instead of SD
**Bolded** = Statistically significant differences between groups in ANOVA analyses
