# Supplementary material for: Pubertal Timing Associates With Cardiometabolic Markers During Puberty and in Young Adulthood
Source: Acta Paediatr. 2026 May 29;115(10):2168–90. doi: 10.1111/apa.70624 (PMC13552584; doi:10.1111/apa.70624)
Supplement: Supplementary file 5 [file APA-115-2168-s005.docx]

**Figure S3.** Visual presentation of mean low density lipoprotein cholesterol (LDL-C) by group. **A)** Beginning of breast development (B2) early, average, late **B)** Beginning of testicular development (G2) early, average, late **C)** Menarche (M) early, average, late **D)** End of testicular development (G5) early, average, late.

| **A** | **B** |
| --- | --- |
| **C** | **D** |

Note: Differences between groups at the pubertal age seen in tables may not be easily interpreted in graphs as ages differ.
