# Supplementary material for: Pubertal Timing Associates With Cardiometabolic Markers During Puberty and in Young Adulthood
Source: Acta Paediatr. 2026 May 29;115(10):2168–90. doi: 10.1111/apa.70624 (PMC13552584; doi:10.1111/apa.70624)
Supplement: Supplementary file 7 [file APA-115-2168-s007.docx]

Table S3. Descriptive values by group of beginning of testicular development (G2)

|  | G2_early_ N= 47 | | | G2_average_ N=105 | | | G2_late_ N=83 | | |
| --- | --- | --- | --- | --- | --- | --- | --- | --- | --- |
| Age at G2 | 10–11 years, 19.4 % | | | 12 years, 44.9 % | | | 13–17 years, 35.7 % | | |
|  | Mean  (SD) | Range | n | Mean  (SD) | Range | n | Mean  (SD) | Range | n |
| At 7 years of age | | | | | | | | | |
| BMI  (kg/m^2^) | 15.9  (1.6) | 13.4–19.9 | 46 | 15.7  (1.5) | 13.0–19.7 | 103 | 15.6  (1.8) | 12.1–10.6 | 81 |
| **Height**  **(cm)** | **126.8**  **(4.3)** | **117.0–134.3** | **46** | **125.3**  **(4.4)** | **114.1–135.5** | **103** | **124.0**  **(5.0)** | **112.8–137.3** | **81** |
| WC  (cm) | 55.3  (3.3) | 50.0–64.0 | 42 | 55.2  (4.0) | 47.0–67.0 | 96 | 55.4  (4.4) | 47.0–67.5 | 73 |
| sBP  (mmHg) | 101.3  (8.4) | 79.0–126.0 | 46 | 100.0  (7.2) | 80.0–127.0 | 103 | 98.1  (8.0) | 76.0–121.0 | 81 |
| dBP  (mmHg) | 60.1  (6.0) | 46.0–71.0 | 46 | 57.9  (6.6) | 40.0–75.0 | 103 | 57.7  (7.7) | 42.0–93.0 | 81 |
| TC  (mmol/L) | 4.4  (0.6) | 3.4–5.8 | 45 | 4.5  (0.7) | 2.6–7.2 | 100 | 4.4  (0.7) | 3.0–6.4 | 79 |
| HDL-C  (mmol/L) | 1.3  (0.2) | 0.8–1.9 | 45 | 1.3  (0.3) | 0.7–2.5 | 100 | 1.3  (0.2) | 0.8–2.1 | 79 |
| LDL-C  (mmol/L) | 2.8  (0.5) | 1.7–3.8 | 45 | 2.9  (0.6) | 1.1–5.2 | 100 | 2.8  (0.6) | 1.7–4.2 | 79 |
| TG*  (mmol/L) | 0.7  (0.5–0.8) | 0.3–2.1 | 45 | 0.6  (0.5–0.8) | 0.2–2.0 | 100 | 0.6  (0.5–0.7) | 0.3–1.2 | 79 |
| At G2 | | | | | | | | | |
| **BMI**  **(kg/m^2^)** | **17.9 (2.2)** | **13.9–23.7** | **47** | **18.1** (**2.7**) | **13.7–27.7** | **105** | **19.2** (**3.5**) | **13.6–29.3** | **83** |
| **Height**  **(cm)** | **149.8**  **(5.7)** | **135.0–161.8** | **47** | **152.9**  **(6.2)** | **139.3–168.2** | **105** | **158.2**  **(8.8)** | **139.3–183.2** | **83** |
| **WC**  **(cm)** | **64.6** (**5.9**) | **54.5–78.5** | **46** | **66.4** (**7.8**) | **55.0–96.0** | **103** | **70.4** (**10.3**) | **55.5–101.5** | **81** |
| sBP  (mmHg) | 107.2 (9.8) | 91.7–135.3 | 47 | 107.1 (9.4) | 83.3–133.3 | 105 | 108.8 (9.0) | 89.0–136.0 | 83 |
| dBP  (mmHg) | 58.6 (6.7) | 48.0–75.0 | 47 | 59.7 (6.3) | 46.5–78.0 | 105 | 59.4 (6.1) | 45.0–75.5 | 83 |
| **TC**  **(mmol/L)** | **4.5** (**0.7**) | **3.3–6.7** | **47** | **4.4** (**0.8**) | **2.1–6.3** | **105** | **4.1** (**0.7**) | **2.2–5.8** | **83** |
| **HDL-C**  **(mmol/L)** | **1.3** (**0.3**) | **0.75–2.2** | **47** | **1.3** (**0.3**) | **0.8–2.1** | **105** | **1.2** (**0.2**) | **0.6–1.7** | **83** |
| LDL-C  (mmol/L) | 2.8 (0.5) | 1.6–4.5 | 47 | 2.7 (0.7) | 0.9–4.6 | 104 | 2.6 (0.6) | 1.2–4.0 | 83 |
| TG*  (mmol/L) | 0.7 (0.5–0.9) | 0.2–2.9 | 47 | 0.7 (0.5–0.9) | 0.2–1.0 | 105 | 0.6 (0.5–0.9) | 0.4–3.3 | 83 |
| Glucose  (mmol/L) | 4.9 (0.3) | 4.1–5.4 | 12 | - |  | 0 | 5.0 (0.4) | 4.4–6.6 | 28 |
| Insulin*  (mU/L) | 6.9 (4.3–7.9) | 2.7–9.8 | 12 | - |  | 0 | 6.6 (4.7–7.9) | 2.7–17.4 | 28 |
| HOMA-IR* | 1.6 (0.9–1.7) | 0.5–1.2 | 12 | - |  | 0 | 1.5 (1.0–1.8) | 0.5–5.1 | 28 |
| At 20 years of age | | | | | | | | | |
| BMI  (kg/m^2^) | 23.2 (4.6) | 17.5–42.3 | 36 | 23.0 (3.0) | 15.3–34.3 | 90 | 22.6 (3.2) | 16.8–31.6 | 68 |
| Height  (cm) | 182.5  (6.2) | 169.3–194.4 | 36 | 181.4  (5.8) | 165.5–192.9 | 90 | 180.5  (6.8) | 166.6–195.9 | 69 |
| WC  (cm) | 81.2 (10.4) | 67.5–125.0 | 36 | 80.6 (7.0) | 63.0–105.0 | 90 | 80.0 (8.4) | 64.0–106.0 | 69 |
| sBP  (mmHg) | 127.6 (13.1) | 100.6–158.7 | 36 | 127.6 (12.3) | 105.3–160.3 | 90 | 124.6 (11.3) | 92.7–148.0 | 69 |
| **dBP**  **(mmHg)** | **68.2** (**7.5**) | **53.0–89.0** | 36 | **66.0** (**7.9**) | **47.0–83.0** | **90** | **63.6** (**8.1**) | **49.5–86.5** | **69** |
| TC  (mmol/L) | 4.0 (0.7) | 2.7–5.4 | 36 | 4.2 (0.7) | 2.0–5.8 | 89 | 4.0 (0.8) | 2.0–6.3 | 67 |
| HDL-C  (mmol/L) | 1.2 (0.3) | 0.6–2.0 | 36 | 1.2 (0.3) | 0.5–2.0 | 89 | 1.2 (0.2) | 0.6–1.8 | 67 |
| LDL-C  (mmol/L) | 2.3 (0.6) | 1.0–3.5 | 36 | 2.5 (0.6) | 0.6–3.8 | 89 | 2.3 (0.7) | 0.8–4.9 | 67 |
| TG*  (mmol/L) | 1.0 (0.7–1.3) | 0.2–2.1 | 36 | 0.9 (0.6–1.2) | 0.3–2.7 | 89 | 0.9 (0.7–1.2) | 0.4–3.3 | 67 |
| Glucose  (mmol/L) | 4.9 (0.4) | 4.3–5.7 | 36 | 4.9 (0.4) | 4.2–6.1 | 89 | 5.0 (0.3) | 4.3–5.7 | 67 |
| Insulin*  (mU/L) | 6.6 (4.9–9.4) | 1.7–26.8 | 36 | 6.0 (4.9–8.1) | 1.9–46.4 | 89 | 6.2 (4.4–8.4) | 1.6–21.5 | 68 |
| HOMA-IR* | 1.4 (1.0–2.2) | 0.4–6.2 | 36 | 1.3 (1.0–1.8) | 0.4–4.3 | 89 | 1.4 (1.0–1.8) | 0.3–4.7 | 67 |
| CRP*  (mg/L) | 0.6 (0.3–1.7) | 0.1–20.0 | 36 | 0.7 (0.3–1.4) | 0.1–42.4 | 89 | 1.0 (0.3–1.7) | 0.13–41.4 | 67 |
| At 26 years of age | | | | | | | | | |
| BMI  (kg/m^2^) | 25.5 (4.1) | 18.4–36.2 | 27 | 25.4 (3.5) | 18.8–34.1 | 61 | 24.1 (3.4) | 17.2–35.1 | 56 |
| Height  (cm) | 182.4  (6.8) | 168.6–194.4 | 27 | 181.7  (6.1) | 166.6–194.3 | 61 | 181.0  (7.4) | 167.4–196.7 | 56 |
| WC  (cm) | 88.3 (10.1) | 74.3–114.9 | 27 | 87.1 (8.8) | 71.1–107.3 | 61 | 84.6 (8.8) | 70.2–108.3 | 56 |
| sBP  (mmHg) | 127.0 (13.2) | 94.3–148.0 | 27 | 127.4 (8.5) | 110.3–146.7 | 61 | 126.6 (9.6) | 102.3–149.3 | 56 |
| dBP  (mmHg) | 75.9 (9.9) | 53.0–103.3 | 27 | 73.7 (7.8) | 56.3–95.0 | 61 | 74.1 (6.5) | 63.0–91.3 | 56 |
| TC  (mmol/L) | 4.5 (0.6) | 3.4–5.5 | 27 | 4.6 (1.0) | 2.7–7.8 | 61 | 4.5 (0.9) | 2.1–7.1 | 56 |
| HDL-C  (mmol/L) | 1.2 (0.3) | 0.6–1.9 | 27 | 1.2 (0.3) | 0.5–1.9 | 61 | 1.2 (0.3) | 0.7–1.8 | 56 |
| LDL-C  (mmol/L) | 2.8 (0.6) | 1.7–3.9 | 27 | 2.9 (0.9) | 1.0–5.7 | 61 | 2.8 (0.8) | 0.6–5.2 | 56 |
| TG*  (mmol/L) | 0.9 (0.7–1.2) | 0.4–3.5 | 27 | 0.9 (0.8–1.3) | 0.4–3.2 | 61 | 1.0 (0.8–1.3) | 0.4–2.7 | 56 |
| Glucose  (mmol/L) | 5.1 (0.6) | 4. 1–6.9 | 27 | 5.1 (0.5) | 4.2–6.2 | 61 | 5.1 (0.5) | 4.0–6.4 | 56 |
| **Insulin***  **(mU/L)** | **8.2** (**5.5–10.8**) | **2.8–77.4** | **27** | **6.5** (**4.8–8.9**) | **2.3–19.8** | **61** | **6.4** (**4.9–8.4**) | **1.8–17.7** | **56** |
| **HOMA-IR*** | **1.8** (**1.3–2.6**) | **0.6–14.1** | **27** | **1.5** (**1.0–2.2**) | **0.4–5.2** | **61** | **1.4** (**1.0–1.9**) | **0.4–4.5** | **56** |
| CRP*  (mg/L) | 0.6 (0.3–2.0) | 0.1–9.5 | 27 | 0.6 (0.3–1.8) | 0.1–32.4 | 61 | 0.7 (0.4–1.6) | 0.1–4.2 | 56 |
| GlycA  (μmol/L) | 1.3 (1.2) | 1.1–1.9 | 27 | 1.3 (0.2) | 1.1–2.1 | 61 | 1.3 (1.1) | 1.0–1.7 | 56 |

* Logarithm converted, median instead of mean, and IQR instead of SD
**Bolded** = Statistically significant differences between groups in ANOVA analyses
